# SUMO1‐conjugation is altered during normal aging but not by increased amyloid burden

**DOI:** 10.1111/acel.12760

**Published:** 2018-04-06

**Authors:** Trayana Stankova, Lars Piepkorn, Thomas A. Bayer, Olaf Jahn, Marilyn Tirard

**Affiliations:** ^1^ Department of Molecular Neurobiology Max Planck Institute of Experimental Medicine Göttingen Germany; ^2^ Max Planck Institute of Experimental Medicine Proteomics Group Göttingen Germany; ^3^ Division of Molecular Psychiatry Department of Psychiatry and Psychotherapy University Medical Center Göttingen (UMG) Göttingen Germany

**Keywords:** 5XFAD, aging, Alzheimer, His_6_‐HA‐SUMO1 knock‐in, SUMOylation

## Abstract

A proper equilibrium of post‐translational protein modifications is essential for normal cell physiology, and alteration in these processes is key in neurodegenerative disorders such as Alzheimer's disease. Recently, for instance, alteration in protein SUMOylation has been linked to amyloid pathology. In this work, we aimed to elucidate the role of protein SUMOylation during aging and increased amyloid burden in vivo using a His_6_‐HA‐SUMO1 knock‐in mouse in the 5XFAD model of Alzheimer's disease. Interestingly, we did not observe any alteration in the levels of SUMO1‐conjugation related to Alzheimer's disease. SUMO1 conjugates remained localized to neuronal nuclei upon increased amyloid burden and during aging and were not detected in amyloid plaques. Surprisingly however, we observed age‐related alterations in global levels of SUMO1 conjugation and at the level of individual substrates using quantitative proteomic analysis. The identified SUMO1 candidate substrates are dominantly nuclear proteins, mainly involved in RNA processing. Our findings open novel directions of research for studying a functional link between SUMOylation and its role in guarding nuclear functions during aging.

## 1 INTRODUCTION

Imbalances in protein homeostasis lead to the misfolding, aggregation and subsequent accumulation of various proteins into large deposits (Hartl, 2017). In the brain, the accumulation of misfolded proteins is part of a physiological process that leads to an age‐related decline in cognitive performance. These dysfunctional processes are accelerated in major neurodegenerative diseases where misfolded amyloid beta, tau, huntingtin, ataxin, or α‐synuclein is thought to cause the synaptic stress and neuronal death observed in Alzheimer's disease (AD), Huntington's disease, ataxia, and Parkinson's disease (Krumova & Weishaupt, 2013). Post‐translational modifications of amyloid beta, tau, huntingtin, ataxin, or α‐synuclein are determinants of their aggregation propensity, their structure, and their conformational state, and abnormal modifications influence the cytotoxic capacity of the corresponding species (Russell, Koncarevic & Ward, 2014).

Analogous to ubiquitination, SUMOylation is a post‐translational protein modification that consists of the attachment of a SUMO moiety to a lysine residue of a target protein. In mammals, three SUMO paralogues can be attached to a target. They are classified into two groups, SUMO1 and SUMO2/3, not only based on their sequence homology but also based on other criteria such as their ability to form chains, their expression levels, and their response to stress (Flotho & Melchior, 2013; Nayak & Muller, 2014). Specifically, in contrast to SUMO2/3, SUMO1 is rarely found as a free moiety in cells. SUMOylation is essential for proper cell growth, function, and signaling, and the cycle of SUMOylation/de‐SUMOylation is highly dynamic and sensitive to multiple regulatory and environmental influences (Chymkowitch, Nguea & Enserink, 2015; Eifler & Vertegaal, 2015; Flotho & Melchior, 2013). SUMO substrates are classically found in the cell nucleus where they regulate various processes such as transcription, DNA replication and repair, chromatin organization, splicing, and ribosome assembly (Chymkowitch et al., 2015; Hendriks et al., 2017). Moreover, in extranuclear compartments, SUMOylation has been proposed to regulate enzymatic activity, ion channel activity, signaling by G protein‐coupled receptors, mitochondrial dynamics, and various cytoskeletal proteins (Alonso et al., 2015; Andreou & Tavernarakis, 2009). Finally, SUMOylation can affect protein stability or localization and is able to change protein–protein interactions, thus regulating the assembly or disassembly of protein complexes (Chymkowitch et al., 2015; Eifler & Vertegaal, 2015; Flotho & Melchior, 2013). For example, the GTPase‐activating protein RanGAP1, which is the most abundant SUMO1 target in cells, relocalizes to the nuclear envelope upon SUMO1 conjugation where it regulates nuclear trafficking (Mahajan, Delphin, Guan, Gerace & Melchior, 1997; Ritterhoff et al., 2016).

In the brain, expression levels of the components of the SUMOylation cycle are high during brain development and remain moderate in the adult brain, with SUMO1 showing a broad distribution in neuronal and glial cells over the entire adult mouse brain (Hasegawa, Yoshida, Nakamura & Sakakibara, 2014; Watanabe, Takahashi, Tomizawa, Mizusawa & Takahashi, 2008). Like in every other cell type studied so far, SUMOylation substrates are dominantly found in the nucleus of neuronal cells, but some controversy exists regarding extranuclear substrates in general and synaptic substrates in particular (Daniel et al., 2017). Nevertheless, SUMOylation has attracted increasing interest in the field of neurosciences and has been shown to regulate neuronal function under physiological and pathophysiological conditions (Krumova & Weishaupt, 2013; Wang et al., 2014; Yang & Paschen, 2015).

The role of SUMOylation is particularly interesting in the context of neurodegenerative disorders with deregulated proteostasis (Feligioni et al., 2015; Krumova & Weishaupt, 2013; Lee, Sakurai, Matsuzaki, Arancio & Fraser, 2013; Liebelt & Vertegaal, 2016). Notably, SUMOylation can regulate the solubility of various disease‐associated proteins (Krumova & Weishaupt, 2013; Lee et al., 2013; Shahpasandzadeh et al., 2014). Furthermore, altered SUMOylation—at the global level of SUMOylated proteins or with regard to the SUMOylation status of specific disease‐related proteins—has been reported in the context of a large variety of neurological disorders with altered proteostasis, including AD as the most common age‐related neurological disorder (Lee et al., 2013; Martins, Tasca & Cimarosti, 2016). Indeed, several AD‐related proteins (such as α‐synuclein, tau, and APP) have been proposed to be SUMO1 conjugates (Martins et al., 2016). Additionally, SUMOylation levels have been correlated to synaptic plasticity and cognitive function in normal physiology and amyloid beta pathology (Lee et al., 2014; Marcelli et al., 2017). Therefore, understanding the role of SUMOylation is particularly interesting not only during age‐related neurodegenerative stress such as AD but also during physiological aging (Andreou & Tavernarakis, 2009; Feligioni et al., 2015; Krumova & Weishaupt, 2013; Liebelt & Vertegaal, 2016).

A major challenge in studying protein SUMOylation is the specific analysis of endogenous substrates. In that context, the use of the His_6_‐HA‐SUMO1 knock‐in (KI) mouse line in combination with high‐affinity antibodies against the HA tag has proven to be a powerful tool for the identification and localization of endogenous SUMO1 conjugates in vivo*,* as well as their enrichment by affinity purification (Daniel et al., 2017; Tirard & Brose, 2016; Tirard et al., 2012). The addition of the His_6_‐HA tag after the start codon of the endogenous *Sumo1* locus does not alter the overall pattern of SUMO1 conjugation as visualized by Western blot, the localization of SUMO1 substrates in vivo, nor the global pool of SUMO1 substrates as identified by mass spectrometry (Becker et al., 2013; Daniel et al., 2017; Tirard et al., 2012). Indeed, lysine acceptor site mutation within SUMO peptides or addition of small tags has been widely used in the SUMO proteomics field with no obvious changes in global SUMOylation capacity (Hendriks & Vertegaal, 2016; Matic et al., 2010), and particularly, the replacement of SUMO by tagged variants is well tolerated in all model organisms tested so far (Kaminsky et al., 2009; Miller, Barrett‐Wilt, Hua & Vierstra, 2010; Panse, Hardeland, Werner, Kuster & Hurt, 2004).

In this study, we made use of the His_6_‐HA‐SUMO1 KI mouse model to test the current hypothesis that links SUMO1 conjugation to alterations in proteostasis during normal aging and amyloid burden. To this end, we used the 5XFAD mouse model that shows clear age‐related AD features such as amyloid deposition, synaptic loss, and age‐related cognitive decline (Oakley et al., 2006). Strikingly, we found age‐related alterations of SUMO1 conjugation in this AD model but did not detect any significant changes in SUMO1 conjugation related to an increased amyloid burden.

## 2 RESULTS

### 2.1 Generation and characterization of double mutant mice His_6_‐HA‐SUMO1::5XFAD

The SUMO1 KI mouse line has been established as useful tool to study *bona fide* SUMO1 substrates (Daniel et al., 2017; Tirard & Brose, 2016; Tirard et al., 2012). Here, we assessed SUMO1 conjugation during alterations of proteostasis, as observed during aging and the development of AD‐like pathology. For this purpose, we crossed the His_6_‐HA‐SUMO1 knock‐in (KI) with the 5XFAD mouse model that rapidly recapitulates major features of AD, including neuronal loss in hippocampal and cortical regions, and age‐dependent synapse loss (Oakley et al., 2006). We generated double mutant mice that are referred to here as KI/AD; non‐KI and non‐AD mice were used as controls, and are referred to as KI/WT, WT/AD, and WT/WT. Immunostaining of amyloid beta using the 6E10 antibody on brain sagittal sections from both KI/AD and WT/AD mice confirmed that the KI/AD mice develop intense intraneuronal amyloid immunostaining, starting from the age of 8 weeks, and extracellular plaques from the age of 8–16 weeks, with kinetics similar to the WT/AD mice (Figure S1a). Additionally, we observed a drastic increase in brain levels of GFAP in old KI/AD as compared to young KI/AD mice (Figure S1b), indicative of gliosis. Together with decreased brain levels of synaptic proteins (data not shown) as described in the 5XFAD mouse model (Oakley et al., 2006), our data indicate that the SUMO1 KI mutation does not change the kinetics and the consequences of amyloidogenesis.

### 2.2 Altered global SUMO1 levels during aging but not during amyloid pathology

**Figure 1:**
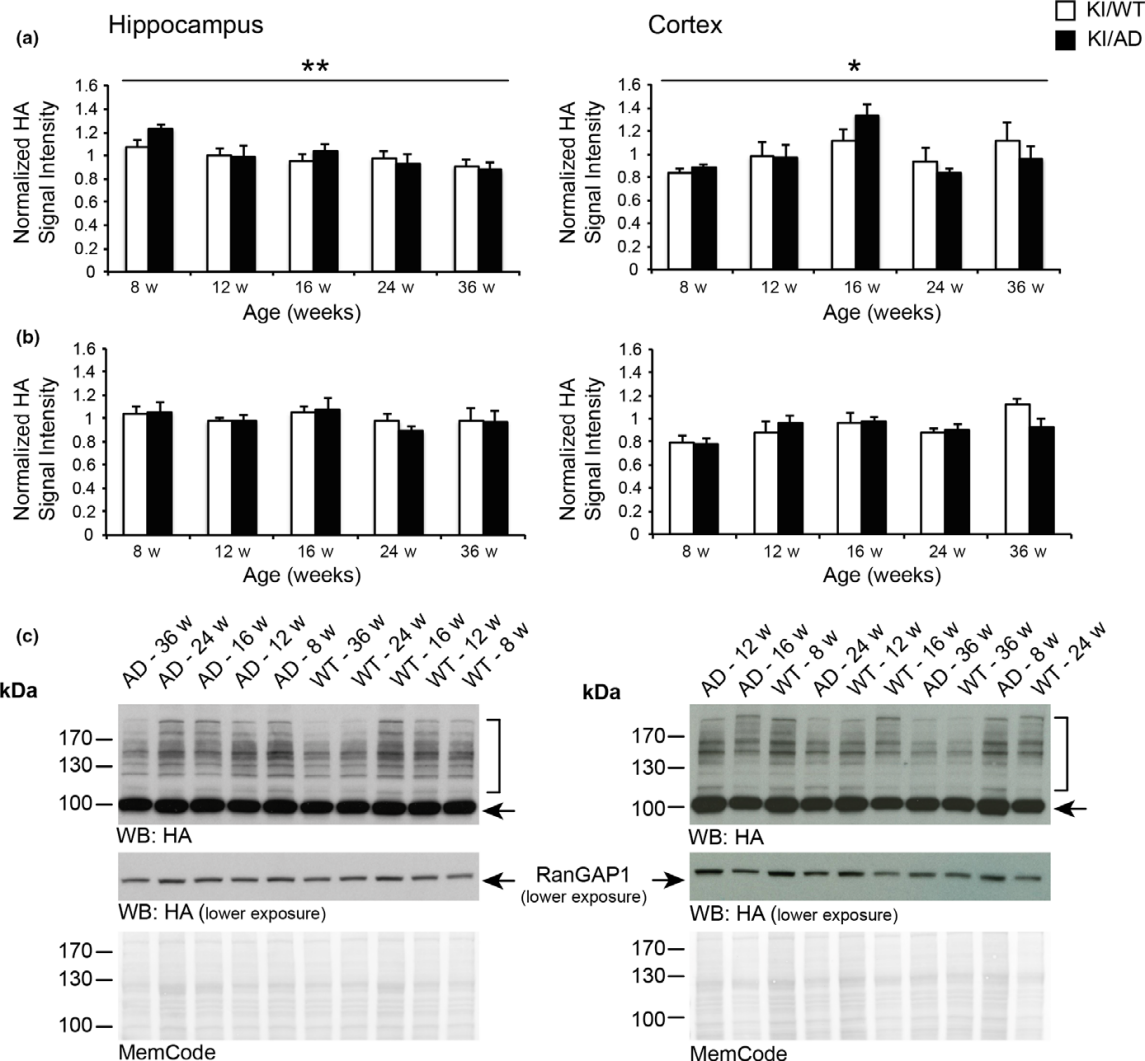
Alterations in SUMO1 conjugate but not RanGAP1 levels during aging. Quantification of SUMO1 conjugate levels (a) and SUMO1‐conjugated RanGAP1 levels (b) in hippocampus (left panels) and cortex (right panels) of KI/WT and KI/AD mice aged 8–36 weeks. Tissues from KI/WT and KI/AD animals were analyzed by SDS/PAGE, blotted on nitrocellulose membranes, stained with total protein stain MemCode followed by anti‐HA staining. Anti‐HA was normalized to MemCode staining and then to the lane average of the analyzed pairs (*N* = 6 animals per age and genotype). Data are expressed as mean ± *SEM* (*N* = 6). Two‐way ANOVA indicated no significant effect of genotype or genotype x age interaction, but age had a significant effect (*p* = .01 for hippocampus samples, *p* = .0169 for cortical samples). (c) Representative Western blots and MemCode stainings. The brackets indicate the anti‐HA signal quantified in the case of global SUMO1 conjugates levels, the arrow indicates SUMO1‐conjugated RanGAP1. All original MemCode and anti‐HA stainings that were used for the analysis, as well as the calculations performed, are included in Datasets S1 and S2

Based on various AD mouse models, several studies indicated changes in global levels of SUMO1 conjugates during amyloid pathology (Lee et al., 2014; Marcelli et al., 2017; McMillan, Brown, Henley & Cimarosti, 2011; Nistico et al., 2014). Accordingly, we tested whether these findings can be recapitulated in our His_6_‐HA‐SUMO1::5XFAD model by making use of the HA tag for high‐affinity detection of SUMO1 conjugates. Using quantitative Western blotting, we assessed global levels of SUMO1 conjugates in cortex and hippocampus of KI/AD as compared to KI/WT animals, in an age range of 8–36 weeks (Figure 1). Within this time window, amyloid beta 1–40 and amyloid beta 1–42 gradually accumulate in 5XFAD mouse brains, and amyloid deposits and gliosis also gradually increase to reach a plateau by the age of 36 weeks, where synapse loss is observed (Oakley et al., 2006).

In Western blot analyses, SUMOylated proteins give a typical signal that consists of a smear of protein bands at high molecular weight (Figure 1c, brackets). In the case of SUMO1, this signal appears above the most abundant SUMO1 target, RanGAP1, the SUMOylated form of which is visible at 90 kDa (Figure 1c, black arrow). Surprisingly, two‐way ANOVA revealed a significant effect of age in both hippocampal and cortical tissues (Figure 1a), but no significant effect of genotype or a genotype x age interaction on SUMO1 conjugate levels, indicating that amyloid burden does not alter global SUMO1 conjugation levels in hippocampus and cortex (Figure 1a,c). In the hippocampus, a small but steady decrease in global SUMO1 conjugate levels was observed between 8 and 36 weeks of age in both KI/WT and KI/AD animals (Figure 1; *p* = .01, *N* = 6). In contrast, in the cortex, a transient increase was observed that peaks at the age of 16 weeks, again in both KI/WT and KI/AD animals (Figure 1, *p* = .0169, *N* = 6). Surprisingly however, hippocampal and cortical levels of SUMOylated RanGAP1 were unaltered during increased amyloid burden and aging (Figure 1b,c). Altogether, with the exception of SUMOylated RanGAP1, these data indicate mild alterations in global SUMO1 levels during aging but not during amyloidogenesis, with different effects in the hippocampus and cortex.

### 2.3 SUMO1 substrates stay primarily nuclear during aging and amyloid pathology

**Figure 2:**
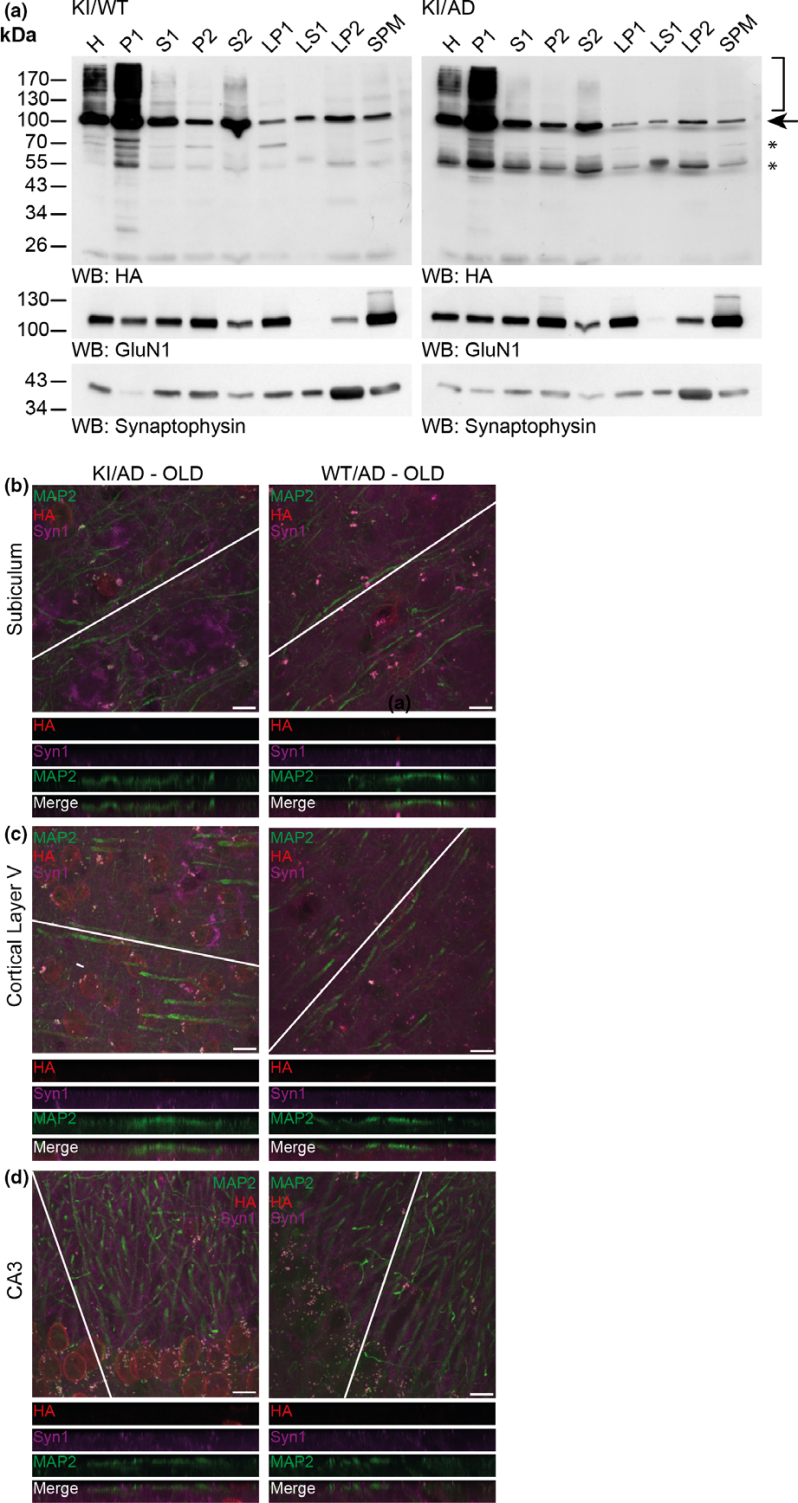
SUMO1 conjugates remain nuclear during increased amyloid burden. (a) Western blot analysis of subcellular fractions of 36‐week‐old KI/WT and KI/AD mouse brain using anti‐HA antibody (upper two panels) and antibodies to GluN1 (a marker of the postsynaptic compartment) and Synaptophysin (a marker of the presynaptic compartment) to validate the fractionation procedure (lower two panels). H, homogenate; P1, nuclear pellet; S1, supernatant after P1 sedimentation; P2, crude synaptosomal pellet; S2, supernatant after P2 sedimentation; LP1, lysed synaptosomal membranes; LS1, supernatant after LP1 sedimentation; LP2, pellet after LS1 sedimentation, SPM, synaptic plasma membranes. Bracket indicates the anti‐HA signal representing SUMOylation, arrow indicates RanGAP1, stars indicate nonspecific signal detected by the anti‐HA antibody. (b) Brain sagittal sections of aged (36 weeks old) KI/AD (left panels) and WT/AD (right panels) mice were immunostained using antibodies directed against HA (red, labels HA‐HA conjugates), MAP2 (green, labels neuronal somata and dendrites), and Synapsin1 (Syn1, magenta, labels synapses). The images show triple‐labeled neurons of hippocampal subiculum (b), cortical layer 5 (c), and proximal apical dendrite from hippocampal CA3 (d). The white line shows the orientation of the line‐scan used to generate the image stack shown in the bottom side view. Scale bar: 10 μm. Note that the anti‐HA immunosignal is mainly located in neuronal nuclei, only background staining is observed in WT/AD mice. Little anti‐HA signal is observed along MAP2‐positive structures and does not colocalize with Synapsin1

Increased amyloid stress during aging leads to synaptic loss (Oakley et al., 2006). Therefore, it is possible that increased amyloid burden alters the subcellular localization of SUMO1 conjugates in extranuclear compartments in general and at synapses in particular. Using biochemical methods and immunostaining, we demonstrated previously that SUMO1 conjugates are mainly localized to the cell nucleus in adult mice aged 8–12 weeks (Daniel et al., 2017; Tirard et al., 2012). Here, we determined whether aging and/or amyloid pathology alters the subcellular distribution of SUMO1 substrates in vivo, for example*,* by increasing the amount of extranuclear SUMO1 targets, particularly with regard to synapses. At first, we performed subcellular fractionation of aged (>36 weeks) KI/AD and KI/WT mouse brains (Figure 2a). Western blot analysis of the various subcellular fractions revealed that the majority of SUMO1 conjugates remained predominantly in the nuclear P1 fraction in both KI/WT and KI/AD mice (Figure 2a, bracket), similar to material from 8‐week‐old animals (Daniel et al., 2017; Tirard et al., 2012). A weak anti‐HA signal was observed in S2 fractions but not in subsequent fractions—in particular not in the synaptic plasma membrane (SPM) fraction—indicating that aging and amyloid burden do not alter the overall distribution of SUMO1 substrates in the brains of aged mice.

SUMOylation is a highly labile and transient modification, and it is possible that SUMO1‐conjugated proteins are lost during the preparation of the SPM fractions. Therefore, we used high‐resolution imaging to determine whether aging and/or amyloid burden alters the localization of SUMO1 conjugates in vivo (Figure 2b–d). We studied anti‐HA immunosignals in three different brains regions: the hippocampal subiculum (Figure 2b), cortical layer 5 (Figure 2c), as these two regions are severely affected by the amyloid pathology in AD mice at a later age, and the hippocampal CA3 (Figure 2d) as it is a region where we previously showed extranuclear but nonsynaptic SUMO1 punctates along MAP2‐positive processes (Tirard et al., 2012).

Firstly, we evaluated how amyloid pathology influences the subcellular distribution of SUMO1 target proteins. We captured *z*‐stack images of neurons in the hippocampal subiculum (Figure 2b, left panel), cortical layer 5 (Figure 2c, left panel), and hippocampal CA3 (Figure 2d, left panel) of aged (36 weeks) KI/AD mice in comparison with aged WT/AD (Figure 2b–d, right panels). Anti‐HA signals were predominantly observed in neuronal nuclei, and only background staining was detected in aged WT/AD (Figure 2b–d, right panel). Line scanning through MAP2‐positive processes of neurons revealed a weak extranuclear HA signal, which did not colocalize with the Synapsin1 signal in all three brain regions studied (Figure 2b–d, bottom side views), indicating that increased amyloid burden did not trigger any visible changes in the subcellular localization of neuronal SUMO1 conjugates *in vivo*.

Next, to determine whether aging alters the subcellular distribution of SUMO1 proteins targets, we analyzed anti‐HA immunostaining of brain sections of young (8 weeks, Figure S2, left panels) and aged (36 weeks, Figure S2, middle panels) KI/WT mice in comparison with aged WT/WT (Figure S2, right panels). Line scanning through MAP2‐positive processes of neurons confirmed our previous observation that the specific extranuclear SUMO1 signal is weak and barely above background level, especially when compared to the HA signal in WT/WT mice that were used as negative controls for anti‐HA staining (Figure S2, right panels). Importantly, SUMO1 signals appeared similar in both ages and remained primarily nuclear, with a dominant localization at the nuclear envelope and within the nuclear area in all the brain regions analyzed (Figure S2a–c). Altogether, our data indicate that the global subcellular localization of SUMO1 substrates remained primarily nuclear and is not altered during aging and amyloid pathology in vivo.

### 2.4 SUMO1 is not detected in amyloid plaques

**Figure 3:**
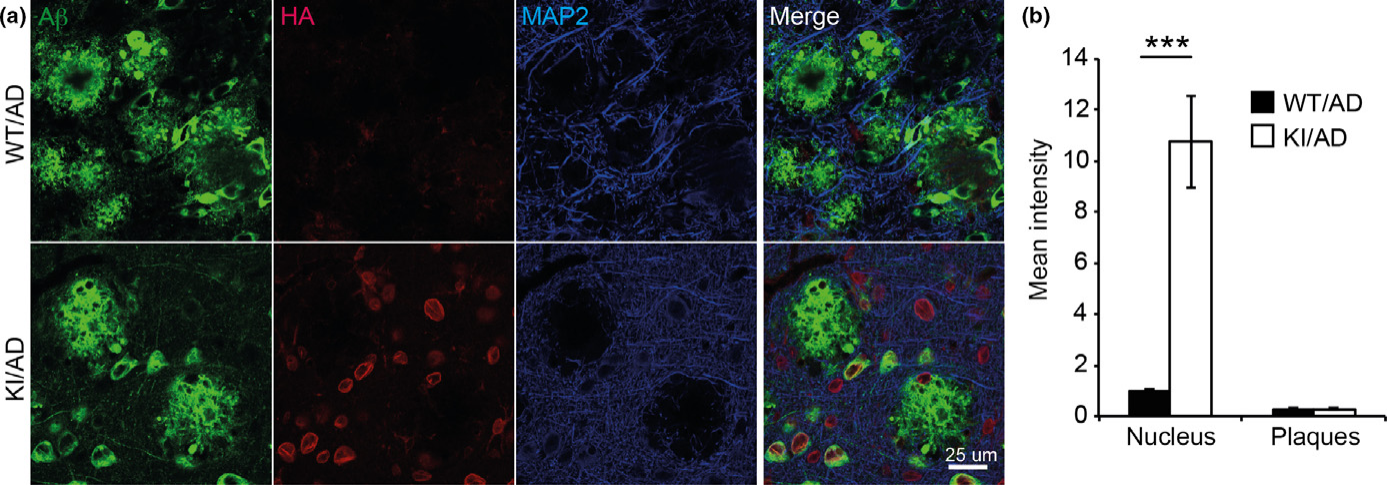
SUMO1 conjugates are not localized to amyloid plaques. (a) Sagittal brain sections of 24‐week‐old KI/AD and WT/AD animals were stained using anti‐HA antibodies (red), 6E10 antibodies (green) that label amyloid beta 1–42 among other amyloid beta variants (epitope lies within amino acids 3–8 of amyloid beta) and anti‐MAP2 antibodies (blue). Sections of the hippocampal subiculum are shown. Images are representatives of three independent experiments. Scale bar, 25 μm. (b) Anti‐HA signal intensity in amyloid plaques and in cell nuclei was quantified using ImageJ (*N* = 3, ***significance between WT/AD and KI/AD,* p* = .0007 in Student's *t* test)

A number of studies alluded to the presence of SUMO1 in amyloid plaques (Lee et al., 2013). We therefore used our mouse model to test this notion. We performed double immunostaining of HA and amyloid beta using the 6E10 antibody in 24 weeks old WT/AD and KI/AD mice, that is, at an age at which many amyloid plaques have accumulated in the hippocampal subiculum (Figure 3a). We quantified anti‐HA signal intensity within intense amyloid‐positive structures in KI/AD mice as compared to WT/AD mice, which served as negative controls for background staining. As internal positive control, the intensity of the anti‐HA staining in cell nuclei of KI/AD mice was measured as compared to WT/AD mice (Figure 3). Intensity levels of the anti‐HA signals at the nuclear envelopes and within nuclei of neurons from the subiculum in KI/AD animals were significantly higher than those in WT/AD animals (Figure 3b, *N* = 3, Student's *t*‐test *p* = .0007). Strikingly, however, in hippocampal subiculum, the intensity of the anti‐HA immunostaining in amyloid plaque structures was similar in both WT/AD and KI/AD mice and comparable to the anti‐HA background intensity levels observed in the nuclei of WT/AD cells, indicating that anti‐HA staining in amyloid plaques is not specific. Therefore, SUMO1 substrates are not found in amyloid plaques in His_6_‐HA‐SUMO1::5XFAD mouse brain.

### 2.5 Analysis of the SUMO1 conjugation status of AD‐related proteins in vivo

**Figure 4:**
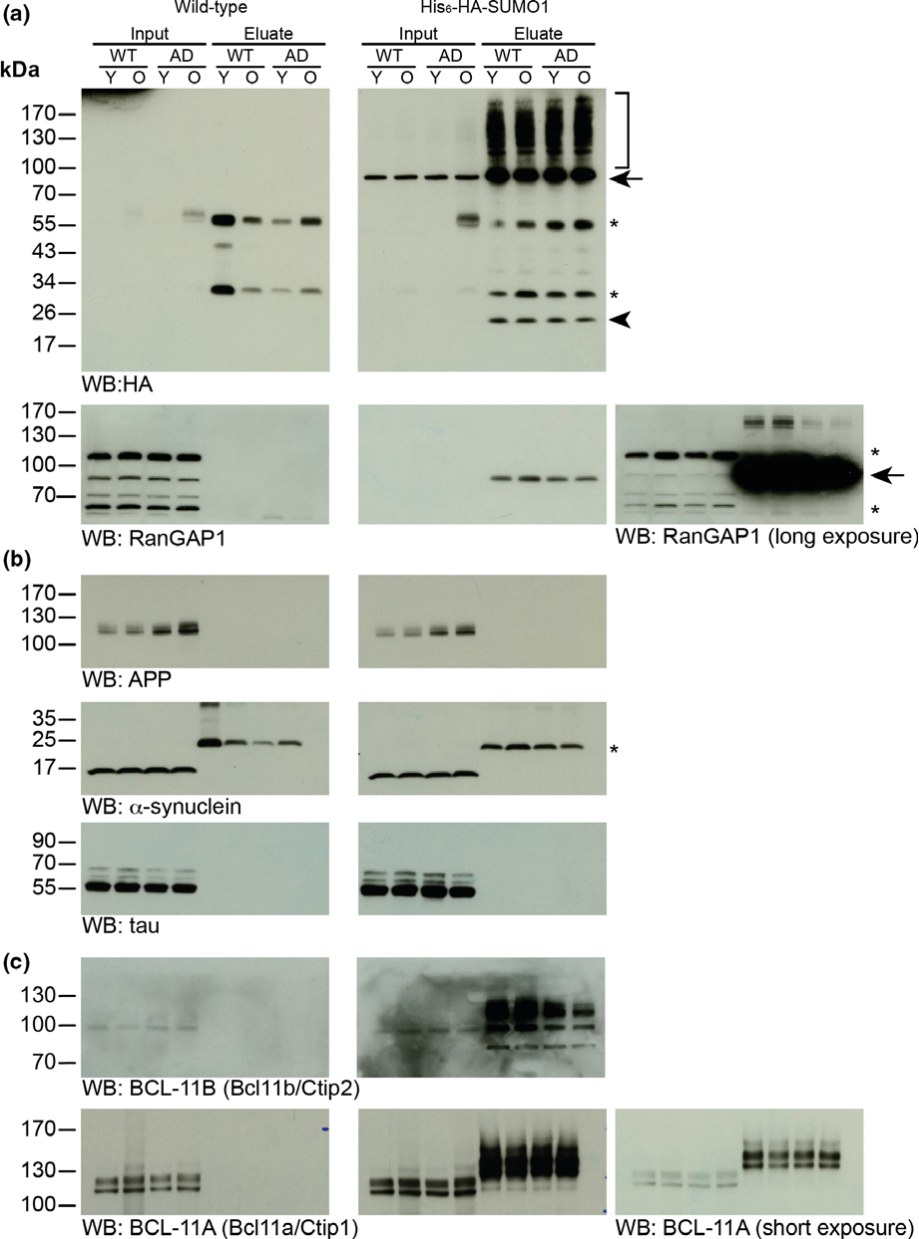
Western blot analysis of SUMO1 conjugates in vivo. Representative detergent extract input sample (Input) from 8‐week‐old (young, Y) and 36‐week‐old (old, O) WT/WT, WT/AD, KI/WT, and KI/AD animals and specific HA‐peptide eluates (Eluate) of HA immunoaffinity‐purified samples were analyzed by SDS/PAGE and Western blotting (WB) with antibodies to the indicated proteins. Black arrows indicate RanGAP1, stars indicate nonspecific bands, arrowhead indicates free SUMO1 peptide, and bracket indicates His_6_‐HA‐SUMO1 conjugates

Several studies indicated that APP, tau, and α‐synuclein are SUMO1 conjugates in vitro (Dorval & Fraser, 2006; Luo et al., 2014; Zhang & Sarge, 2008). Therefore, we investigated whether these findings can be confirmed in vivo using the His_6_‐HA‐SUMO1 KI mouse model and HA‐based affinity purification methods (Tirard & Brose, 2016). We performed anti‐HA immunoprecipitation (IP) from total brain of young (8 weeks) and aged (36 weeks) KI/WT and KI/AD animals, and used age‐matched WT/WT and WT/AD samples as negative control for the IP (Figure 4). Anti‐HA Western blot analysis of input (Figure 4, left panels) and anti‐HA affinity‐purified material (Figure 4, right panels) revealed a strong enrichment of His_6_‐HA‐SUMO1 conjugates specifically in KI samples while only background was observed in WT samples (Figure 4a). Accordingly, the SUMO1 substrate RanGAP1 was strongly enriched solely in KI samples (Figure 4a, indicated by an arrow). Western blot analysis of input and anti‐HA IP eluate using anti‐APP, α‐synuclein, and tau antibodies did not reveal an enrichment of their corresponding SUMOylated species (Figure 4b), indicating that in our mouse model, APP, α‐synuclein, and tau are either not SUMOylated or their SUMOylation is too transient or low to be detectable in vivo. Another reason for the absence of detectable SUMOylated tau and α‐synuclein might be that the AD mice model we used does not favor the formation of tau‐containing fibrillary tangles or α‐synuclein aggregates.

### 2.6 In vivo Identification of candidate SUMO1 substrates related to AD and aging

Surprisingly so far, our results indicated alterations in global SUMO1 conjugation levels during normal aging but not during amyloid pathology. Furthermore, we found that the localization of SUMO1 conjugates remains unchanged during aging and AD pathology and that SUMO1 conjugates are not found in amyloid plaques. Therefore, it appears unlikely that alterations in the SUMO1 conjugation equilibrium contribute significantly to any toxic or neuroprotective processes related to AD (Martins et al., 2016). However, as the level of SUMOylated RanGAP1 did not decrease during aging as seen for global SUMO1 conjugation, we cannot exclude the possibility that subtle changes involved in aging or AD progression can occur at the level of individual (especially nuclear) SUMO1 substrates. With the aim of gaining insights into SUMOylation networks related to physiological aging and increased amyloid pathology, we engaged in a quantitative proteomic approach to globally assess SUMO1 conjugation and to identify putative individual SUMO1 targets relevant for aging and AD.

We performed anti‐HA‐based affinity purification from young (8 weeks) and old (36 weeks) mouse brains of all genotypes—WT/WT, WT/AD, KI/WT, and KI/AD—followed by label‐free quantification of purified proteins by liquid chromatography coupled to mass spectrometry (LC‐MS). Two brains per condition were processed, and technical replicates were performed at the level of in‐solution digestion, resulting in 32 LC‐MS analyses from which 1,580 proteins were quantified in total (Table S1, worksheets “all proteins pre‐imputation” and “all proteins post‐imputation”). For the identification of specifically affinity‐enriched proteins (and thus of candidate SUMO1 substrates), protein abundance values in the merged KI group were compared to those in the merged control group using a two sample *t* test and a volcano plot with stringent threshold criteria in the Perseus computational platform. From the pool of significantly different proteins (Table S1, worksheet “*t* test KI vs. CTRL”), only the 130 proteins with a positive difference (i.e., which are up‐regulated in KI) were considered further (Figure S3; Table S1, worksheet “enriched in KI”). As many as 11 *bona fide* SUMO1 substrate candidates known from previous screens, including RanGAP1, as the most prominent example, appeared as highly enriched in KI (Figure S3; Table S1, worksheet “enriched in KI,” highlighted in red (Becker et al., 2013; Tirard et al., 2012)). This finding, together with the Western blot confirmation of SUMO1 conjugation performed for RanGAP1 as well as BCL‐11A (Bcl11a/Ctip1) and BCL‐11B (Bcl11b/Ctip2), validated our proteomic approach (Figure 4a,c).

SUMO substrates often belong to large protein complexes (Hendriks et al., 2017). Thus, we subjected the 130 KI‐enriched candidates to network analysis, using the Search Tool for the Retrieval of Interacting Genes/Proteins (STRING) at a high confidence (Table S2, Figure S4a). Forty‐two percent of the identified proteins (55 of 130) appeared to be interconnected with an average local clustering coefficient of 0.346, indicating potential identification of secondary interaction‐dependent non‐SUMOylated proteins and confirming that SUMOylation occurs on highly interconnected networks (Figure S4a; Hendriks et al., 2014). Subsequent analysis of the molecular interaction networks with Cytoscape revealed two main core clusters with scores between 9 and 6, with one of the cluster containing proteins of the SUMOylation machinery (Figure S4b). The other cluster contains proteins functionally related to protein translation indicating a putative regulatory role of SUMOylation in regulating the synthesis of proteins (Figure S4b).

**Figure 5:**
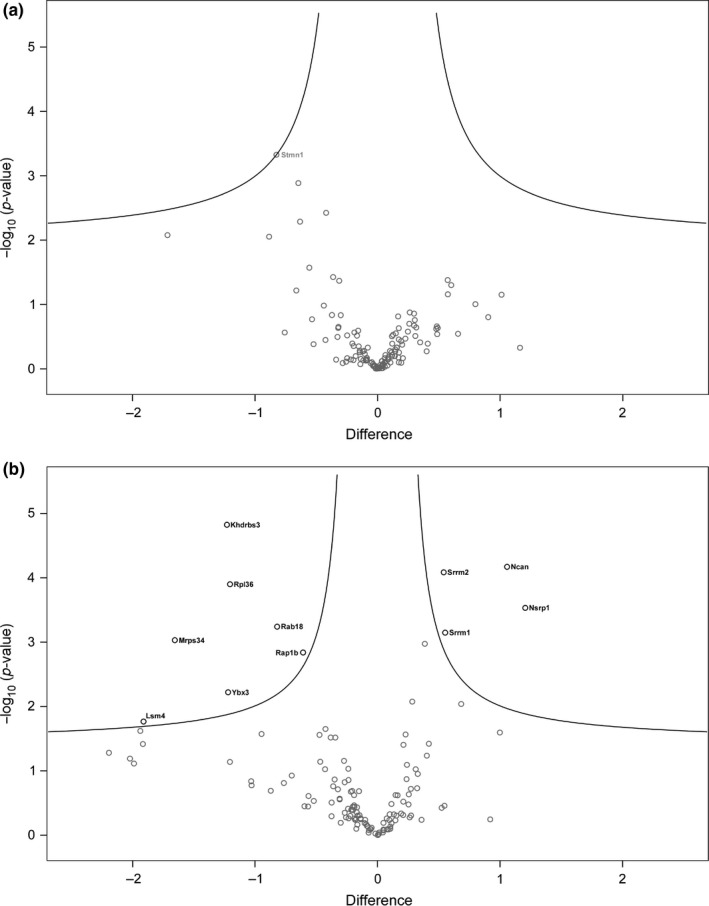
Volcano plots of the 130 KI‐enriched SUMO1 candidate substrates. (a) Disease‐based comparison. No protein appeared to have altered SUMO1 conjugation. Stmn1 is labeled as the candidate closest to statistical significance. (b) Age‐based comparison. Eleven proteins appeared to have altered SUMO1 conjugation (gene names marked in black). In comparison with the two sample *t* test for the identification of KI‐enriched proteins (Figure S3), significance criteria were lowered here (s0 = 0.1, FDR = 0.05) to prevent initial exclusion of candidates with only very subtle changes

Next, we analyzed the proteomic data of the 130 KI‐enriched proteins as to whether they would reflect alterations in SUMO1 conjugation during amyloid burden and/or aging. Interestingly, it was observed that the overall median of the log2 abundance values is lower by 0.29 units in old compared to young KI mice, while no such consistent trend was apparent when KI/AD was compared with KI/WT or when the comparisons were drawn among the respective control conditions (Table S1, worksheet “enriched in KI”). Notably, the log2 abundance values of RanGAP1 did not show a decrease with age, but appeared rather stable over all conditions. Taken together, mass spectrometric protein identification indicated a trend toward a global decrease in SUMO1 conjugation in aging mice independent of amyloid burden, thereby reinforcing our observations from immunoblotting, at least in the hippocampus (Figure 1). For the purpose of identifying individual SUMO1 targets relevant for aging and/or AD, we used the statistical tools in Perseus to analyze changes related to the disease genotype without considering age and changes related to age without considering the disease genotype (Table S1, worksheets “*t* test WT vs. AD” and “*t* test 8w vs. 36w,” respectively). As before, data analysis by two sample *t* test and volcano plot was used, but less stringent threshold criteria were applied to prevent initial exclusion of candidates with only very subtle changes. Interestingly, in the disease‐based comparison, protein abundances appeared unaltered overall (Table S1, worksheet “*t* test WT vs. AD”; Figure 5a). In contrast, the age‐based comparison revealed 11 candidates, which appeared to be significantly altered (Table S1, worksheet “*t* test 8w vs. 36w”; Figure 5b, highlighted in black). These findings from the proteomic approach are essentially in line with our observations so far, indicating that major changes in SUMO1 conjugation occur during aging rather than during increased amyloid burden, even though we cannot exclude that these changes reflect age‐related variation in the expression levels of the candidates. In view of the 11 proteins potentially reflecting age‐related changes in SUMOylation, our proteomic data indicated an increase in SUMO1 conjugation with age for four candidates (Nsrp1, Ncan, Srrm1, and Srrm2) and a decrease in SUMO1 conjugation with age for seven candidates (Khdrbs3, Rpl36, Mrps34, Rab18, Ybx3, Lsm4, and Rap1b). Interestingly, in comparison with the former group, identification of the candidates from the latter group was based on much more robust proteomic data as reflected by a more than fourfold higher average sequence coverage (Table S3). Thus, we considered the proteins with age‐related decrease as the more likely SUMOylation substrates, whereas we considered the proteins with age‐related increase as somewhat ambiguous, in particular Ncan due to its extracellular localization. Taken together, the finding that the significantly down‐regulated targets were higher in number and in sequence coverage was in agreement with our initial observation of a global decrease in SUMO1 conjugation in aging mice.

**Figure 6:**
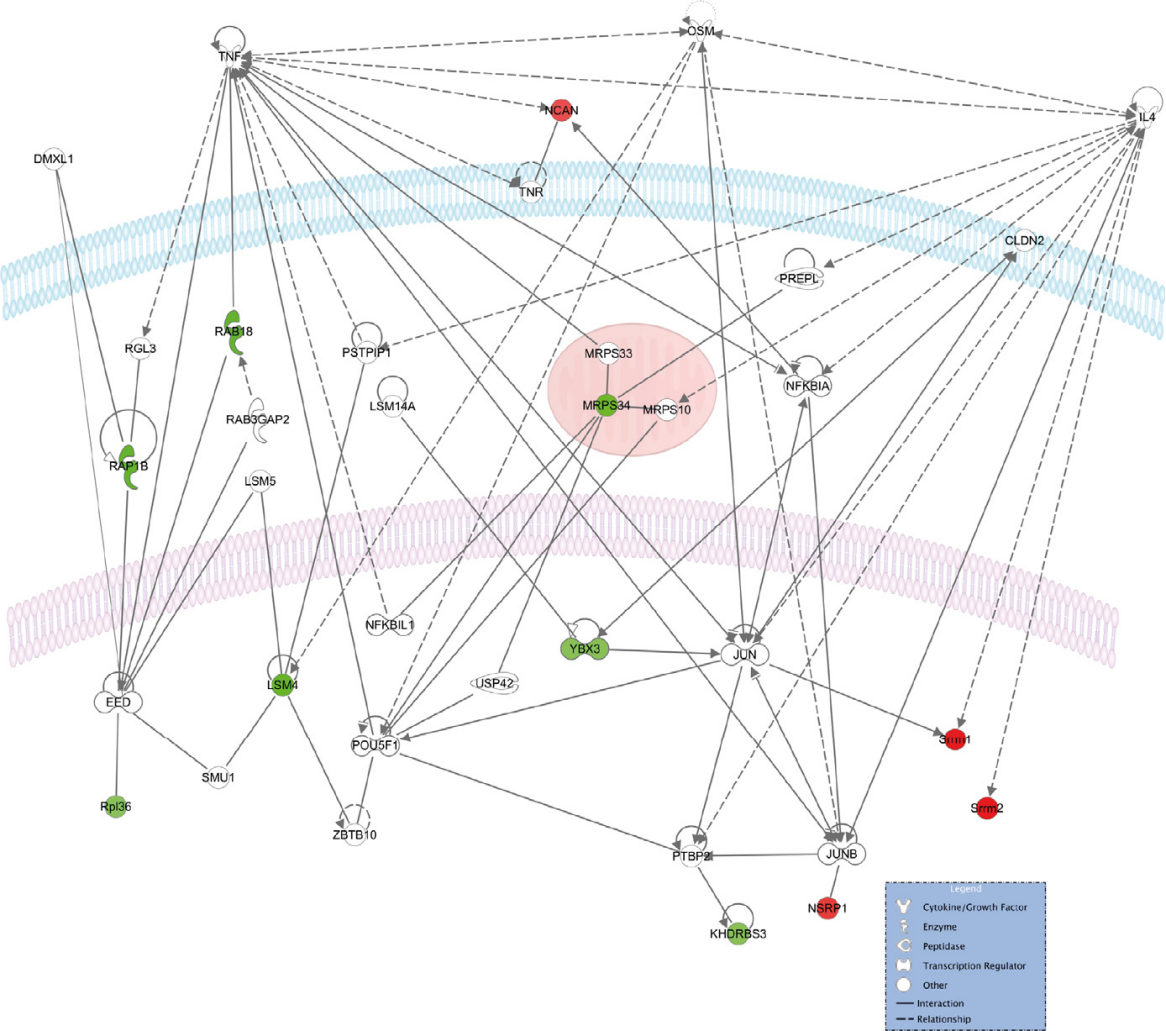
Network visualization using Ingenuity Pathway Analysis of the 11 SUMO1 candidate substrates altered with age. Identified proteins with increased abundance are in red, and identified proteins with decreased abundance are in green. Cellular membrane is blue, nuclear membrane is in light purple, mitochondria are in red

Core expression analysis of all candidates with age‐related alterations in SUMO1 conjugation using Ingenuity Pathway Analysis (IPA) revealed that the identified proteins are mainly involved in RNA processing mechanisms (Table S2, worksheet “Disease and Function”), and one network was associated with the 11 identified candidates (Table S2, worksheet “IPA Network Age”). Visualization of this network revealed that the identified proteins belong to strongly interconnected signaling pathways (Figure 6). When focusing on SUMO1 candidate targets down‐regulated with age, these proteins were found to be dominantly localized to the nucleus (Khdrbs3, Ybx3, Lsm4, and Rpl6) and are mainly involved in RNA processing pathways (Khdrbs3, Ybx3, Lsm4), highlighting a putative role for SUMO1 in regulating RNA processing during aging. Although somewhat less prominent, also ribosomal proteins (Rpl36 and Mrps34) and Ras‐related proteins (Rab18, Rab1b, and Rab3GAP2) are connected to this network, indicating a potentially interesting role for SUMO1 conjugation in protein folding and Ras signaling during age‐related neuronal stress (Figure 6).

Altogether and very surprisingly, our data indicate that SUMO1 conjugation is more relevant during neurodevelopment than during increased amyloid burden. Further studies will be required, especially at later ages, to determine how these changes translate into age‐related neuronal loss and alterations of cognitive functions.

## 3 DISCUSSION

Altered SUMOylation has been correlated to neurological disorders with altered proteostasis in general and to amyloid beta pathology in AD in particular. Using His_6_‐HA‐SUMO1 KI mice crossbred with the 5XFAD mouse model of AD, we here assessed the in vivo effects of increased amyloid burden on the levels and localization of SUMO1 targets in comparison with normal aging. Surprisingly, our data indicate that changes in SUMO1 conjugation are more pronounced, and hence likely more relevant, during brain development and aging than in the context of increased amyloid burden. While we found no significant correlation of altered brain SUMOylation with the AD‐related 5XFAD mutation, we observed alterations in the levels but not the cellular distribution of brain SUMO1 conjugates during normal aging, indicating that SUMOylation may play an important role in the aging process.

### 3.1 SUMOylation and amyloid burden

Variation in global SUMO1 levels in the context of increased amyloid burden was reported in different AD mouse models (Lee et al., 2014; McMillan et al., 2011; Nistico et al., 2014). For instance, in young (3–6 months old) Tg2576 animals, SUMO1 conjugate levels in hippocampal and cortical tissues were found to be increased, while SUMO1 levels at later stage (17 months old) were comparable to those of age‐matched control animals (Marcelli et al., 2017; McMillan et al., 2011; Nistico et al., 2014). In 18‐month‐old 5XFAD animals (Yun et al., 2013), only unconjugated SUMO1 level was found to be increased in cortical tissues as compared to age‐matched controls, possibly reflecting SUMO1 deconjugation.

In the 5XFAD model, AD‐like pathology starts to develop around 2 months of age, and accumulation of amyloid plaques, gliosis, synaptic loss, and memory deficit reach a peak by the age of 9 months (Oakley et al., 2006). This time course of disease development in the 5XFAD model therefore allows the study of early‐stage and late‐stage AD‐like pathology. Accordingly, we analyzed global SUMO1 conjugation levels in 5XFAD and control mice from 2 to 9 months of age. Strikingly, we did not detect any significant link between global SUMO1 levels and AD‐like pathology. This finding argues against an involvement of altered SUMO1 conjugation in the pathogenesis of AD, at least as it is apparent in the 5XFAD model, but does not exclude a link to SUMO2/3 conjugation in this model, as SUMO2/3‐conjugation is prone to react more robustly to stress (Bernstock et al., 2018; Liebelt & Vertegaal, 2016).

Based on immunostaining approaches, previous studies yielded apparent evidence for the presence of SUMO1 in amyloid plaques in 8‐month‐old 5XFAD mice (Yun et al., 2013) and in 16‐month‐old APP transgenic Swedish/PS1ΔE9 mice (Cho et al., 2015). However, the anti‐SUMO1 antibodies used in the corresponding studies did not detect the typical strong signal in the nuclear envelope, which is due to the most abundant SUMO1 substrate, RanGAP1 (Lee et al., 1998; Matunis, Wu & Blobel, 1998). This indicates that the antibodies used in these studies were unsuitable for the specific and sensitive detection of SUMO1 (Daniel et al., 2017). In our study, which was based on the highly specific detection by Western blotting and immunostaining of endogenous SUMO1 via an engineered HA tag in the His_6_‐HA‐SUMO1 KI, it was revealed that the localization of SUMO1 and its targets is not altered during normal aging or by increased amyloid burden. Furthermore, our data indicate that SUMO1 is not present in amyloid plaques or at synapses, although we cannot unequivocally exclude the possibility of a subtle enrichment in plaques that might have been below the detection limit of our methodological approach.

### 3.2 SUMOylation and aging

Currently, information on changes of neuronal SUMOylation levels during normal aging is scarce (Marcelli et al., 2017; McMillan et al., 2011; Nistico et al., 2014). One previous study correlated increased levels of unconjugated SUMO3 with impaired learning ability during aging, but it remained unclear how SUMO2 and SUMO3 were distinguished and SUMO1 levels were not addressed at all (Yang et al., 2012). Interestingly, one study described decreased SUMO1 and Ubc9 mRNA levels in cortices of wild‐type mice aged 3–15 months (Akar & Feinstein, 2009), contrasting with another study describing increased Ubc9 protein expression levels without changes in mRNA levels in non‐AD mouse brains within the same age range (Nistico et al., 2014). Differences in the genetic background of the wild‐type mice used in both studies may account for the conflicting results. More recently, a study showed increased levels of SUMO1 and SUMO2/3 in cortices of wild‐type mice aged 6–10 months as compared to 2‐month‐old animals (Ficulle, Sufian, Tinelli, Corbo & Feligioni, 2018), with a peak in the level of SUMOylated RanGAP1 at the age of 6 month, which is in contrast to our finding in cortical tissues (Figure 1). Differences in the antibodies used to detect SUMO1 conjugates between both studies may account for the divergent trend of changes in SUMO1 levels (Daniel et al., 2017). Unfortunately, a comparison with levels of hippocampal SUMO1 and SUMO2/3 is not possible as those were not analyzed in that study.

Interestingly, our data indicate alterations in the levels of SUMO1 conjugates during aging. A small but steady age‐dependent decline of the levels of SUMO1 conjugates was observed in hippocampal tissue, whereas a transient increase was observed in cortical tissue at the age of 16 weeks. These data represent the first indication of divergent alterations in SUMO1 levels between two different brain regions. Indeed, changes in SUMO1 levels described so far followed similar pattern in hippocampal and cortical tissues (McMillan et al., 2011; Nistico et al., 2014). The reason as to why changes in SUMO1 levels differ between hippocampus and cortex will need to be studied further.

We used a quantitative proteomic approach to complement our analysis of alterations in SUMO1 conjugate levels in general and to identify individual SUMO1 candidate substrates potentially linked to AD or aging in particular. As proof of principle, we found that the SUMO1 candidates identified by anti‐HA‐based affinity purification followed by mass spectrometric protein quantification were also reported in previous proteomic screens (Table S1, KI‐enriched Sheet), indicating that a large portion of the SUMO1 proteome remains stable, irrespective of the physiological context (Becker et al., 2013; Tirard et al., 2012). Many of the identified candidates are transcriptional regulators (Bcl11a/Ctip1, Bcl11b/Ctip2, Wiz, SmchD1, Trim28), indicating that the main function of SUMO1 conjugation is to guard nuclear functions, as may be particularly the case during age‐related proteostasis stress (Gartner & Muller, 2014; Hendriks & Vertegaal, 2016; Liebelt & Vertegaal, 2016).

Globally, mass spectrometric protein quantification indicated a decreased SUMO1 conjugation in aging mice independent of amyloid burden, resembling our observations from immunoblotting (Figure 1a). Consistent with this initial finding, our statistical analysis revealed a small group of candidate SUMO1 targets whose SUMOylation in vivo appeared to be altered during aging, but no SUMO1 candidate was identified to be altered during increased amyloid burden. Interestingly, these age‐related proteins are components of cellular signaling pathways with roles mainly in RNA processing, but also in protein folding and Ras signaling, many of which are known to play a role during aging (Balchin, Hayer‐Hartl & Hartl, 2016; Liu, Cali & Lee, 2017; Longo, 2004; Wang et al., 2011). Most of the nuclear proteins identified as age‐related SUMO1 candidate proteins are involved in the regulation of gene expression, chromatin remodeling, RNA processing, and protein folding. Alterations in the proper function of these processes have been implicated in the modulation of memory and synapse plasticity during aging (Cookson, 2012; Narciso et al., 2016). As SUMOylation is a key regulator of gene expression, it will be very exciting to study whether SUMO1 conjugation may play a role in the development or even in the compensation of age‐related cognitive decline via buffering alterations in DNA damage response and RNA processing (Massaad & Klann, 2011).

## 4 MATERIALS AND METHODS

### 4.1 Animals and ethics statement

Mice were maintained under a controlled environment of 20–25°C, 12/12‐hr light/dark cycle, and 50%–70% humidity, with free access to water and food. All animal procedures were approved by the local government of Lower Saxony. Permits were granted by LAVES Niedersachsen (33.9‐42502‐04‐13/1359). All surgery was performed under isoflurane anesthesia, and all efforts were made to minimize animal suffering.

### 4.2 Subcellular fractionations

Subcellular fractionations were prepared essentially as described previously (Daniel et al., 2017; Jones & Matus, 1974; Tirard et al., 2012). Using glass‐Teflon homogenizer (900 rpm, 12 strokes), brains were homogenized in 10 ml of 320 mm sucrose containing 4 mm HEPES pH 7.4, conventional protease inhibitors (1 μg/ml aprotinin, 0.5 μg/ml leupeptin, 17.4 μg/ml phenylmethylsulfonyl fluoride (PMSF)), and in addition 20 mm N‐ethylmaleimide (NEM) to suppress de‐SUMOylation of proteins by irreversible inhibition of cysteine peptidases. Brain homogenates (H) were centrifuged at 1,000 *g* for 10 min at 4°C in an SS‐34 rotor (Sorvall). The supernatant (S1) was removed from the pellet (P1) and centrifuged at 12,500 *g* for 15 min at 4°C in an SS‐34 rotor. The supernatant (S2) was removed completely, and the synaptosome‐enriched pellet (P2) was resuspended in 9 volumes of cold water containing 4 mm HEPES pH 7.4 and homogenized using a glass‐Teflon homogenizer (1,500 rpm, 10 strokes). The homogenized P2 fraction was centrifuged for 20 min at 4°C in an SS‐34 rotor at 25,000 *g*. The resulting SN (LS1) was ultracentrifuged at 200,000 *g* for 2 hr at 4°C to generate fractions LP2 and LS2. The pellet (LP1) was resuspended in 1 ml of homogenization buffer and layered on top of a two‐step sucrose density gradient (5 ml of 1.2 m and 5 ml of 0.8 m sucrose, 4 mm HEPES, protease inhibitors as above). The gradient was centrifuged at 62,000 *g* for 120 min at 4°C in an SW‐41Ti rotor (Beckman). Synaptosomes were recovered at the interface of 0.8 and 1.2 m sucrose using a Pasteur pipette. The resulting fraction was diluted twofold in water and then pelleted using an SS‐34 rotor at 4°C for 20 min at 37,000 *g* and is referred to as SPM. The various brain fractions are designated as follows: H, homogenate; P1, nuclear pellet; S1, supernatant after P1 sedimentation; P2, crude synaptosomal pellet; S2, supernatant after P2 sedimentation; LP1, lysed synaptosomal membranes; LS1, supernatant after LP1 sedimentation; LP2, pellet after LS1 sedimentation; SPM, synaptic plasma membranes.

### 4.3 Quantitative Western blot

Mice were quickly killed by cervical dislocation. Hippocampus and cortex were carefully removed on ice. Brain regions were lysed in 150 mm NaCl, 1% Triton‐X100, 20 mm Tris pH 7.4 with protease inhibitors (1 μg/ml aprotinin, 0.5 μg/ml leupeptine, 17.4 μg/ml PMSF, and 20 mm NEM). Protein concentration was determined using the BCA assay (Pierce). Protein samples were separated by SDS‐PAGE using precast 4%–12% Bis‐Tris gradient gels (Invitrogen). Transferred proteins were visualized on the membrane using the Pierce total protein stain assay (MemCode, Thermo Fisher). Western blots were probed using anti‐HA antibody (Biolegend) and developed using enhanced chemiluminescence (GE Healthcare) as the signals were too weak for near‐infrared detection. MemCode and anti‐HA sample values were determined using ImageJ. Each sample value was normalized to the MemCode total protein loading value for the corresponding lane and then normalized to the average sample pair value. Samples were loaded three times on various positions on the gel; *N* = 6 mice.

### 4.4 Statistical analysis of quantitative Western blot

Two‐way ANOVA statistical analysis followed by Bonferroni post‐tests were conducted for both hippocampal and cortical tissues using PRISM version 5.0a (GraphPad Software) with genotype and age as factors. No significant main effect of genotype or genotype × age interaction was observed. The age factor was significant for both tissues (hippocampus *p* = .01, cortex *p* = .0169).

### 4.5 Immunohistochemistry

Immunohistochemistry was performed as described (Daniel et al., 2017; Tirard & Brose, 2016; Tirard et al., 2012). Briefly, mice were anesthetized and transcardially perfused with 4% (w/v) paraformaldehyde (PFA) in 0.1 m phosphate buffer (PB), pH 7.4 at 4°C for 10 min. Brains were removed and postfixed for 1 hr at 4°C. The tissue was cryoprotected in 30% (w/v) sucrose in phosphate‐buffered saline (PBS). Sagittal 35‐μm sections were prepared with a cryostat and collected free‐floating in PBS. For immunohistochemistry, sections were pre‐incubated for 1 hr in PBS containing 5% normal goat serum (NGS) and 0.3% Triton X‐100 and then incubated at 4°C for 24 hr in primary antibodies diluted in PBS containing 2% NGS and 0.3% Triton X‐100. After washing repeatedly in PBS, sections were incubated for 2 hr in dye‐coupled secondary antibodies, repeatedly washed, and mounted on slides with Aqua‐Poly/Mount (Polysciences). The antibodies used are listed below.

### 4.6 Primary and secondary antibodies

#### 4.6.1 Primary antibodies

Mouse monoclonal anti‐amyloid beta 1‐16 (6E10, Covance, SIG‐39320, IHC, 1:1,000), mouse monoclonal anti‐APP (Millipore, MAB348, clone 22C11, WB, 1:1,000), chicken anti‐MAP2 (Novus, NB300‐213, IHC, 1:1,000), mouse monoclonal anti‐HA (16B12, Biolegend, 901515, WB, 1:1,000), goat polyclonal anti‐HA (Novus, NB600‐362, IHC, 1:1,000), rabbit polyclonal anti‐RanGAP1 was a kind gift of Frauke Melchior (WB, 1:1,000), mouse monoclonal anti‐Ctip1 (Abcam, ab19487, WB, 1:1,000), rat monoclonal anti‐Ctip2 (Abcam, ab18465, WB, 1:1,000), mouse anti‐GluN1 (Synaptic Systems, 114011, WB, 1:1,000, RRID:AB_887750), mouse anti‐synaptophysin (Synaptic Systems, 101011, WB, 1:1,000, RRID:AB_887824), mouse monoclonal anti α‐synuclein (BD Biosciences, 610786, WB, 1:1,000), mouse monoclonal anti‐Tau (Millipore, MAB3420, WB, 1:2,000), guinea pig polyclonal anti‐GFAP (Synaptic systems, 173 004, WB, 1:1,000), rabbit anti‐Synapsin1/2 (Synaptic Systems, 106002, ICC: 1/2,000, RRID:AB_887804).

#### 4.6.2 Secondary antibodies

HRP‐conjugated goat anti‐mouse (Bio‐Rad, 172‐1011, WB, 1:5,000, RRID:AB_11125936), HRP‐conjugated goat anti‐rabbit (Bio‐Rad, 172‐1019, WB, 1:5,000, RRID:AB_11125143), goat anti‐chicken Alexa Fluor 633 (Thermo Fischer, A‐11039, IHC, 1:1,000, RRID:AB_2534096), goat anti‐mouse Alexa Fluor 488 (Life, A11029, IHC, 1:1,000), donkey anti‐goat Alexa Fluor 555 (Mobitec, IHC, 1:1,000, RRID:AB_2535850).

### 4.7 Imaging and quantification using ImageJ

Single optical sections and *z*‐stacks were acquired at a magnification of 40× (sections) on a confocal laser‐scanning microscope (Leica LSM SP5). During acquisition, imaging parameters (gain and offset) were kept constant for a given labeling and/or genotype to allow for fluorescence intensity comparisons.

Immunosignal quantification was performed using ImageJ. Briefly, images were thresholded and regions of interest (nuclei and/or plaques) were manually selected and signal intensity was quantified. The signal intensity in a defined region was then normalized to the region area to generate the mean intensity. The mean intensity of WT/AD nuclei was set as 1; *N* = 3.

### 4.8 Anti‐HA immunopurification

Anti‐HA affinity purification and Western blot analysis were performed as previously described (Tirard & Brose, 2016; Tirard et al., 2012). For anti‐HA immunoaffinity purification, frozen brains were reduced to powder in a liquid nitrogen bath using a porcelain mortar and pestle. The powder was resuspended in cold RIPA buffer (150 mm NaCl, 1% Triton X‐100, 0.5% sodium deoxycholate, 0.1% SDS, 10 mm Tris, pH 7.6) containing protease inhibitors (1 μg/ml aprotinin, 0.5 μg/ml leupeptine, 17.4 μg/ml PMSF) and 20 mm NEM, sonicated, and ultracentrifuged at 100,000 × *g* for 1 hr at 4°C. The resulting supernatant was passed over a column containing 0.4 ml anti‐HA beads (SIGMA) for 12 hr at a flow rate of 1 ml/min. The column was washed with 100 column volumes of RIPA buffer, and bound material was eluted twice, once at 30°C and once at 37°C, with three column volumes of RIPA buffer containing HA peptide (0.5 mg/ml) synthesized in‐house. Eluates were pooled, and proteins were precipitated as described (Wessel & Flugge, 1984). For Western blotting, the precipitates were dissolved in SDS‐PAGE sample buffer.

### 4.9 Label‐free protein quantification

#### 4.9.1 Proteolytic digestion

Precipitated proteins were dissolved in lysis buffer (7 m urea, 2 m thiourea, 10 mm DTT, 2% CHAPS, 0.1 m Tris pH 8.5) and processed according to a filter‐aided sample preparation (FASP) protocol modified essentially as described by Distler, Kuharev, Navarro and Tenzer (2016). Unless stated otherwise, all steps were automated on a liquid‐handling workstation equipped with a vacuum manifold (Freedom EVO 150, Tecan) using an adaptor device constructed in‐house. Briefly, protein samples were lysed and reduced by shaking for 30 min at 37°C and subsequently loaded on centrifugal filter units (30 kDa MWCO, Millipore). After removal of the detergents by washing twice with wash buffer (8 m urea, 10 mm DTT, 0.1 m Tris pH 8.5), remaining free cysteine residues were alkylated with 50 mm iodoacetamide in 8 m urea/0.1 m Tris pH 8.5 (20 min at RT), followed by two washes with wash buffer to remove excess reagent. Buffer was exchanged by washing three times with 50 mm ammonium bicarbonate (ABC) containing 10% acetonitrile. After three additional washes with 50 mm ABC/10% acetonitrile, which were performed by centrifugation to ensure quantitative removal of liquids, proteins were digested overnight at 37°C with 500 ng trypsin in 40 μl of the same buffer. Tryptic peptides were recovered by centrifugation followed by two additional extraction steps with 40 μl of 50 mm ABC and 40 μl of 1% trifluoroacetic acid (TFA), respectively. Aliquots of the combined flow‐through were spiked with 10 fmol/μl of yeast enolase 1 tryptic digest standard (Waters Corporation) for quantification purposes (Silva, Gorenstein, Li, Vissers & Geromanos, 2006) and directly subjected to LC‐MS analysis.

#### 4.9.2 LC‐MS analysis

Nanoscale reversed‐phase UPLC separation of tryptic peptides was performed with a nanoAcquity UPLC system equipped with a Symmetry C18 5 μm, 180 μm × 20 mm trap column and a HSS T3 C18 1.8 μm, 75 μm × 250 mm analytical column maintained at 45°C (Waters Corporation). Injected peptides were trapped for 4 min at a flow rate of 8 μl/min 0.1% TFA and then separated over 120 min at a flow rate of 300 nl/min with a gradient comprising two linear steps of 3%–35% mobile phase B in 105 min and 35%–60% mobile phase B in 15 min, respectively. Mobile phase A was water containing 0.1% formic acid while mobile phase B was acetonitrile containing 0.1% formic acid. Mass spectrometric analysis of tryptic peptides was performed using a Synapt G2‐S quadrupole time‐of‐flight mass spectrometer equipped with ion mobility option (Waters Corporation). Positive ions in the mass range *m*/*z* 50–2,000 were acquired with a typical resolution of at least 20,000 FWHM (full width at half maximum), and data were lock mass corrected postacquisition. Analyses were performed in the ion mobility‐enhanced data‐independent acquisition mode with drift time‐specific collision energies as described in detail by Distler et al. (2014, 2016). Continuum LC‐MS data were processed for signal detection, peak picking, and isotope and charge state deconvolution using Waters ProteinLynx Global Server (PLGS) version 3.0.2 (Li et al., 2009). For protein identification, a custom database was compiled by adding the sequence information for yeast enolase 1 and porcine trypsin to the UniProtKB/Swiss‐Prot mouse proteome and by appending the reversed sequence of each entry to enable the determination of false discovery rate (FDR). Precursor and fragment ion mass tolerances were automatically determined by PLGS 3.0.2 and were typically below 5 ppm for precursor ions and below 10 ppm (root mean square) for fragment ions. To account for the fact that cysteine residues can be modified by addition of NEM or by carbamidomethylation, both modifications were specified as variable modification in addition to oxidation of methionine. One missed trypsin cleavage was allowed. Minimal ion matching requirements were two fragments per peptide, five fragments per protein, and one peptide per protein. The FDR for protein identification was set to 1% threshold.

#### 4.9.3 Experimental design and data analysis

Of the eight conditions to be compared (WT/WT, WT/AD, KI/WT, and KI/AD with two time points each), affinity‐purified proteins from two individual animals per condition were processed as digestion replicates, resulting in a total of 32 LC‐MS runs. The freely available software ISOQuant (http://www.isoquant.net) was used for postidentification analysis including retention time alignment, exact mass and retention time (EMRT) and ion mobility clustering, data normalization, isoform/homology filtering, and calculation of absolute in‐sample amounts for each detected protein according to the TOP3 quantification approach (Distler et al., 2014, 2016; Kuharev, Navarro, Distler, Jahn & Tenzer, 2015). Only peptides with a minimum length of seven amino acids, which were identified with scores above or equal to 5.5 in at least two runs, were considered. FDR for both peptides and proteins was set to 1% threshold, and only proteins represented by at least two peptides were quantified using the TOP3 method and reported in the ISOQuant output (Table S3). Peptides with variable modifications were excluded from being selected as one of the three most abundant tryptic peptides for TOP3 quantification. Proteins without available gene names (mainly Ig fragments) and contaminants such as cuticular keratins and serum proteins (Alb, Hb) were removed prior to statistical analysis.

For statistical analysis and visualization of the quantification data, protein abundance values in amol were log2‐transformed and imported into the Perseus computational platform (Tyanova et al., 2016). Missing values were imputed using a downshifted normal distribution (width of 0.3 and downshift of 1.8 standard deviations). To identify and visualize specifically enriched proteins on the basis of a maximal number of data points (16 vs. 16), KI group and control group were compared in a group‐wide manner using a two sample *t* test and a volcano plot with a cutoff curve that accounts for a minimal fold change (s0) and a permutation‐based correction for multiple hypothesis testing (FDR). Group‐wide comparison was considered feasible as the means of the individual subgroups consisting of four replicates and the mean of the condition group consisting of 16 replicates were found to be similar. The protein population identified as KI‐specific was then used to evaluate the effects of amyloid burden (independent of age) and of aging (independent of disease state) using the same statistical tools.

The mass spectrometry proteomics data have been deposited to the ProteomeXchange Consortium (http://proteomecentral.proteomexchange.org) via the PRIDE partner repository (Vizcaíno et al., 2016) with the dataset identifier PXD009166.

### 4.10 STRING, Cytoscape, and Ingenuity Pathway Analysis

The known protein–protein interactions within each dataset were obtained from the Search Tool for the Retrieval of Interacting Genes/Proteins (STRING) database (Franceschini et al., 2013) using the KI‐enriched candidates (Table S1). Enrichment analysis was performed allowing network interactions at high confidence (*p* > .7, Table S2, Figure S4) and imported in Cytoscape (Shannon et al., 2003). MCODE analysis was performed as previously described (Hendriks, D'Souza, Chang, Mann & Vertegaal, 2015). Only the proteins with known interactions within the datasets were exported and visualized (Figure S4). The Ingenuity Pathway Analysis software (Qiagen) was used to identify enriched biological functions related to the identified age‐related SUMO1 candidate proteins (Table S2).

## AUTHORS' CONTRIBUTION

MT conceived the project. TAB, OJ, and MT designed the experiments. TS, OJ, and MT performed experiments, acquired, and analyzed the data. MT and OJ wrote the manuscript with the support from TAB and TS.

## CONFLICT OF INTEREST

The authors declare that they have no conflict of interest.
