# Corrigendum

**DOI:** 10.1111/acel.13053

**Published:** 2020-01-21

Stankova, T, Piepkorn, L, Bayer, TA, Jahn, O, Tirard, M. SUMO1‐conjugation is altered during normal aging but not by increased amyloid burden. *Aging Cell*. 2018; 17:e12760. https://doi.org/10.1111/acel.12760

In the article “SUMO1‐conjugation is altered during normal aging but not by increased amyloid burden”, the authors would like to correct the funding information of their article. It should be written as follows:

## Funding information

This work was funded by grants from the Alzheimer Forschung Initiative (AFI DE‐15009) and the German Research Foundation (SFB 1286/A9).

The authors would like to apologize for the inconvenience caused.

