# Supplementary material for: SUMO1‐conjugation is altered during normal aging but not by increased amyloid burden
Source: Aging Cell. 2018 Apr 6;17(4):e12760. doi: 10.1111/acel.12760 (PMC6052395; doi:10.1111/acel.12760)

Supplementary Figure 1

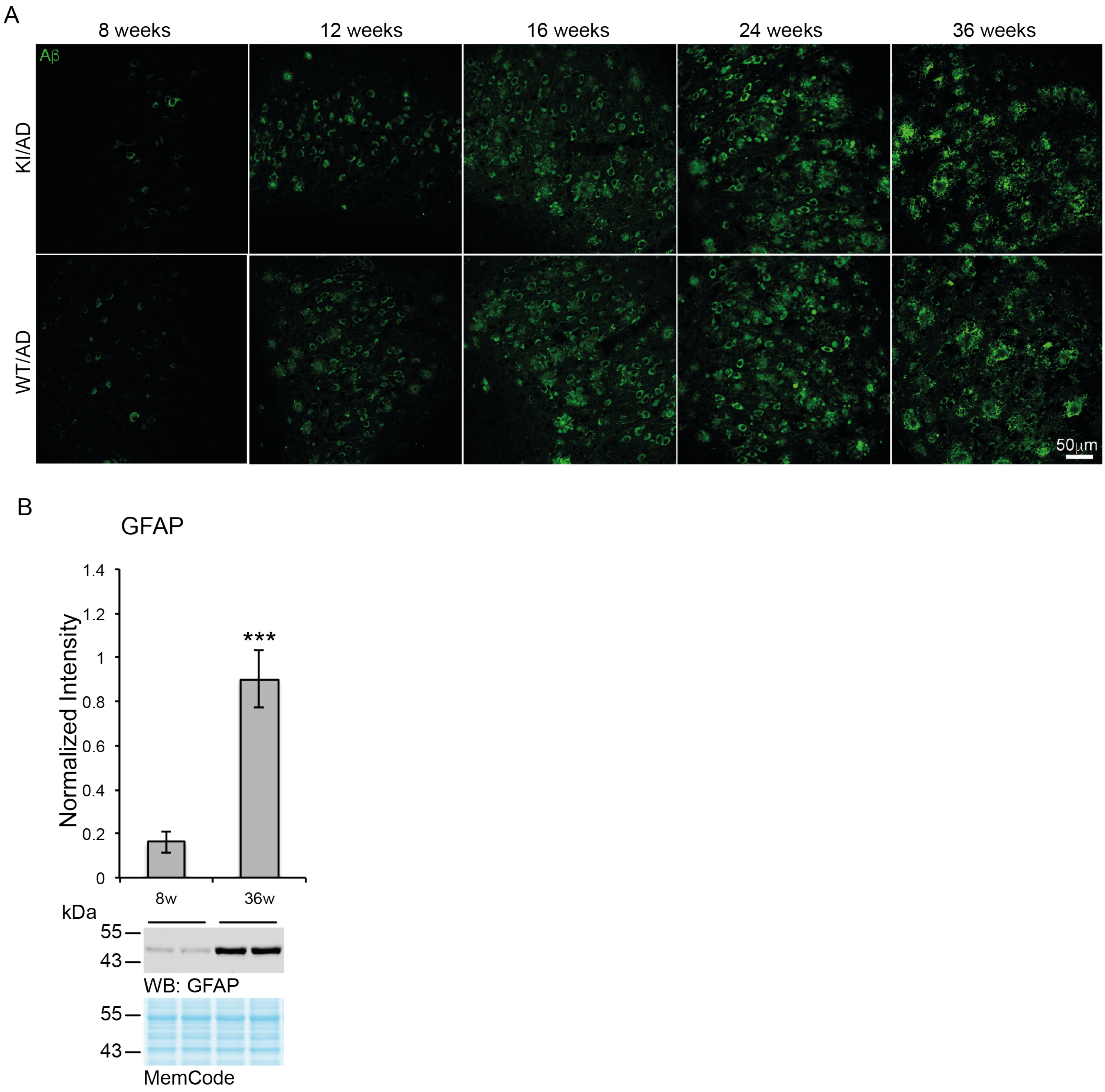

Supplementary Figure 2

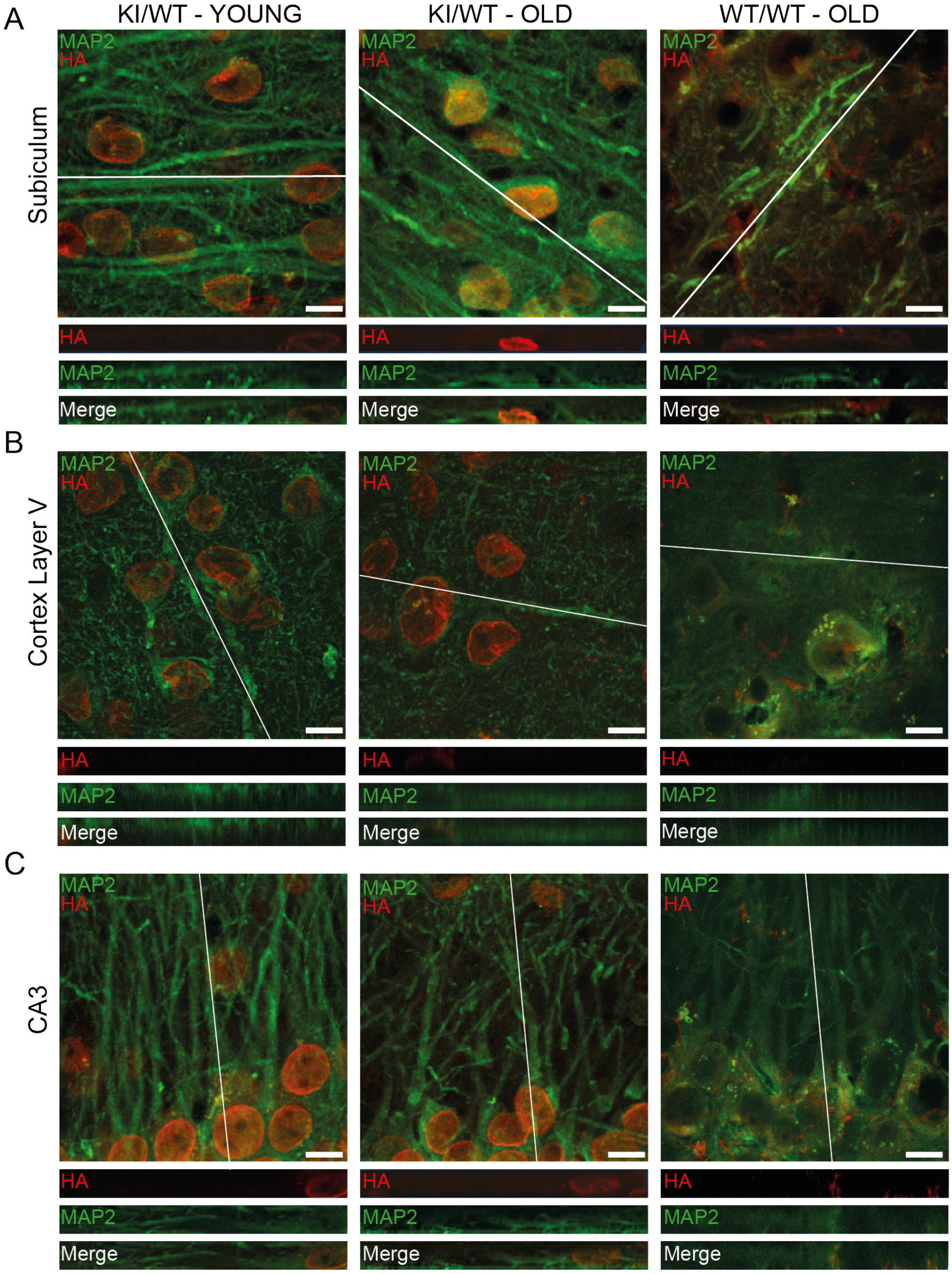

Supplementary Figure 3

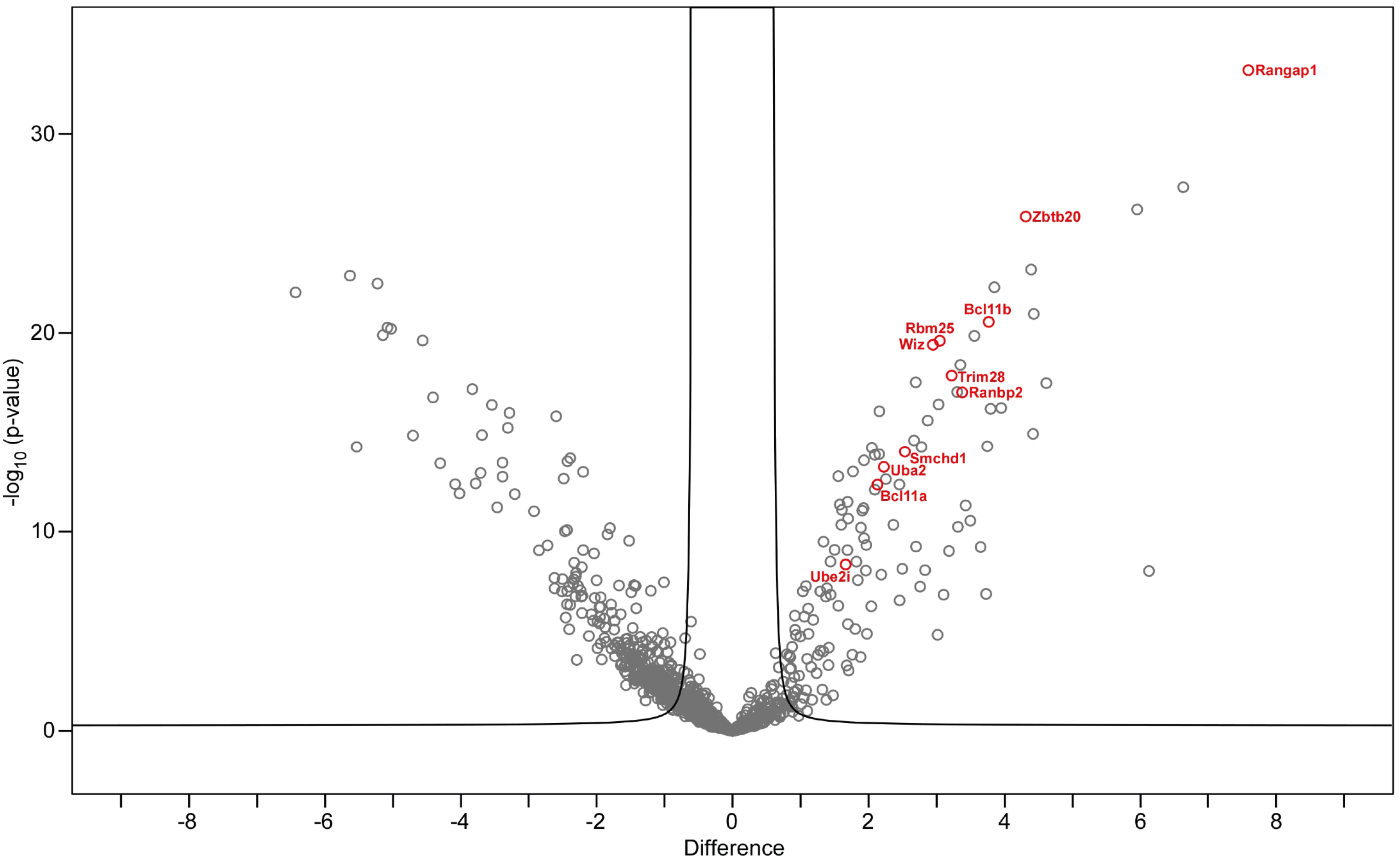

Supplementary Figure 4

A

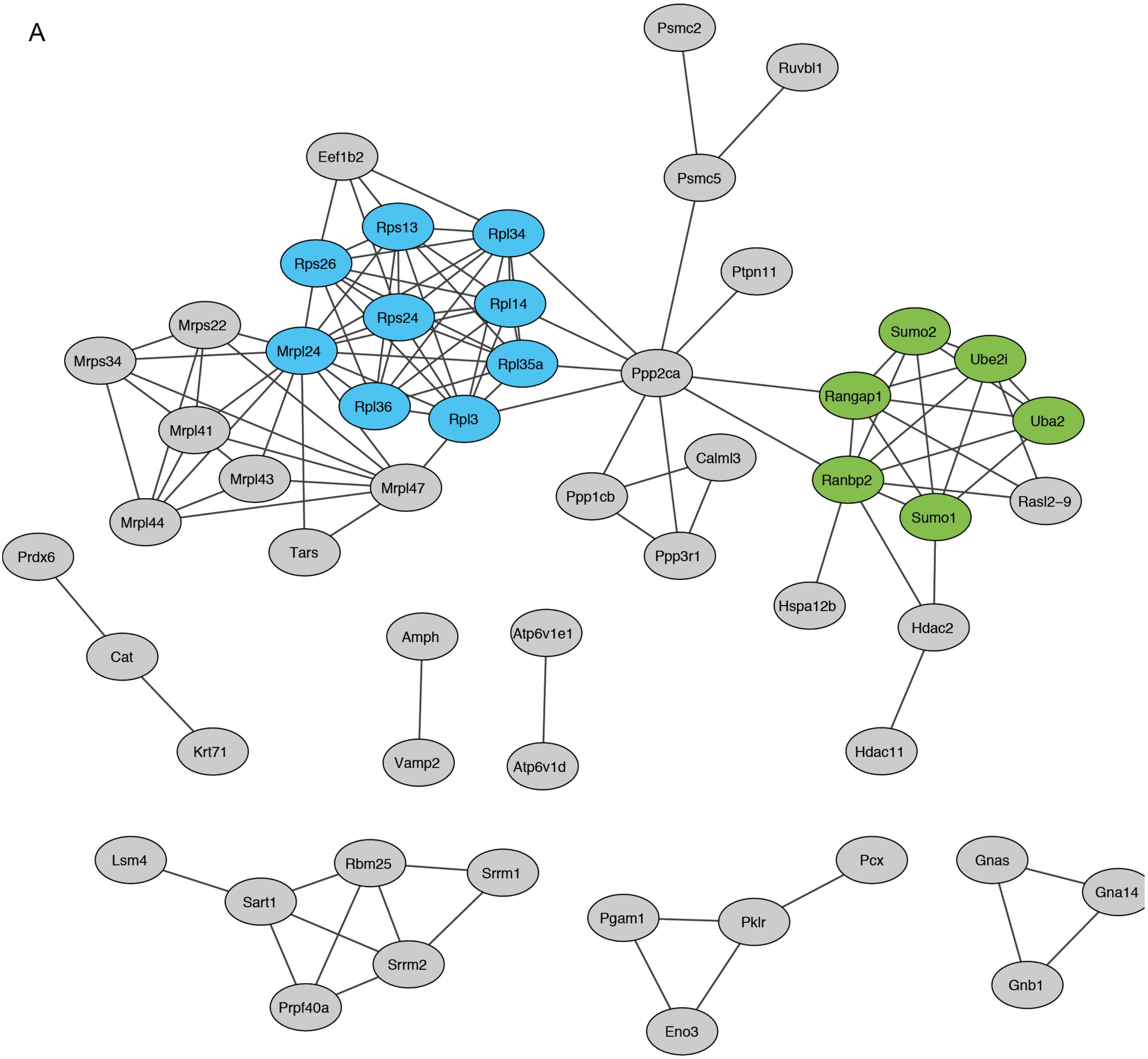


B

Cluster 1: score 9
(Ribosome)

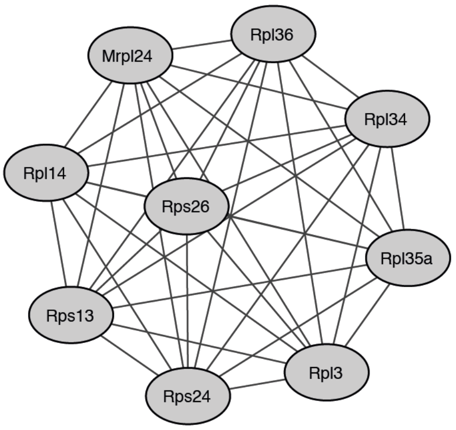


Cluster 2: score 6
(SUMOylation pathway)

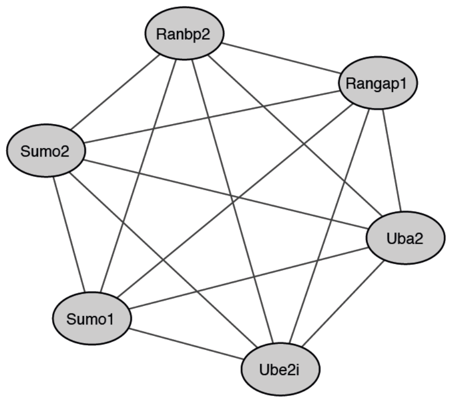

Supplement: Supplementary file 1 [file ACEL-17-na-s001.pdf]
