# Supplementary material for: SUMO1‐conjugation is altered during normal aging but not by increased amyloid burden
Source: Aging Cell. 2018 Apr 6;17(4):e12760. doi: 10.1111/acel.12760 (PMC6052395; doi:10.1111/acel.12760)

Hippocampal Loading I

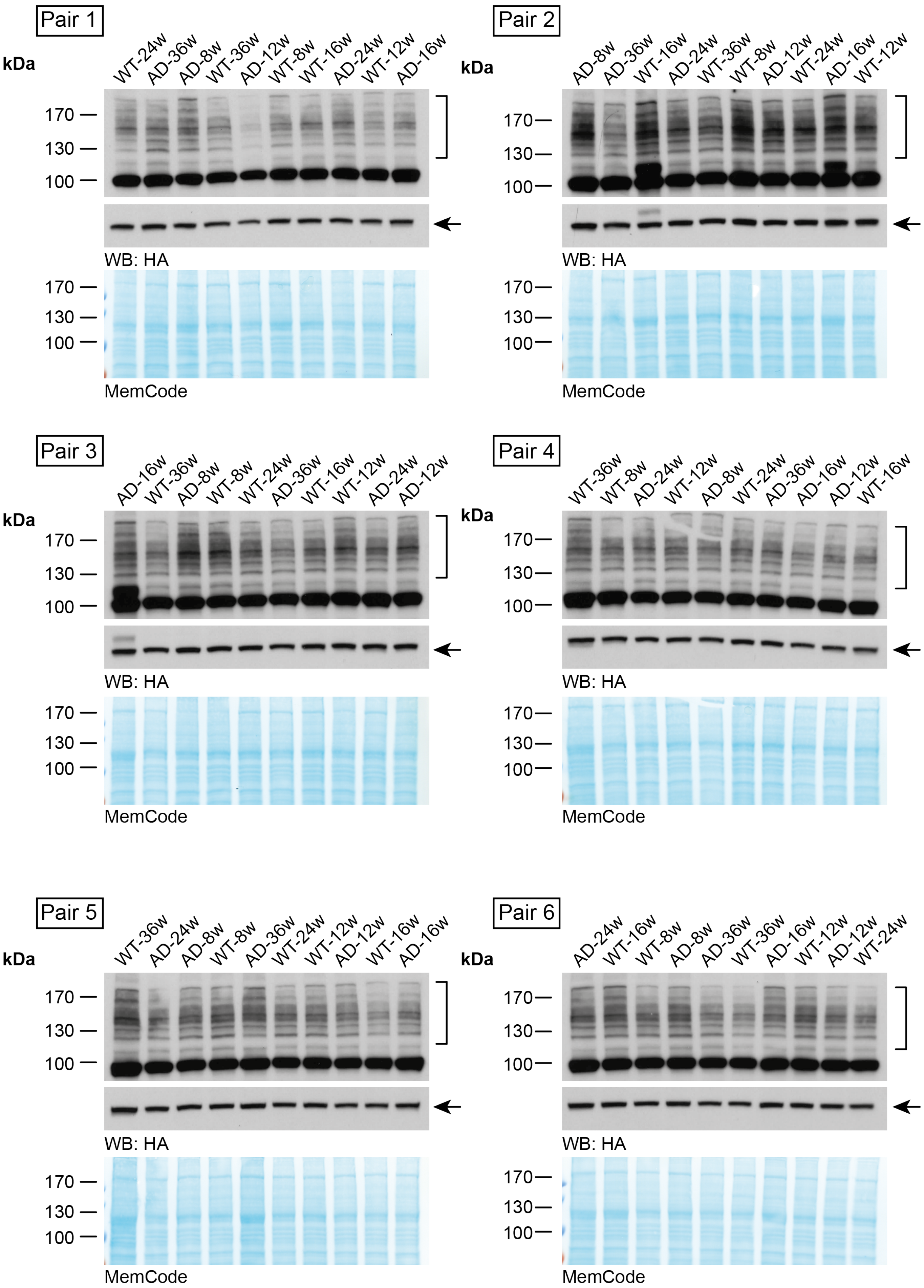

Hippocampal Loading II

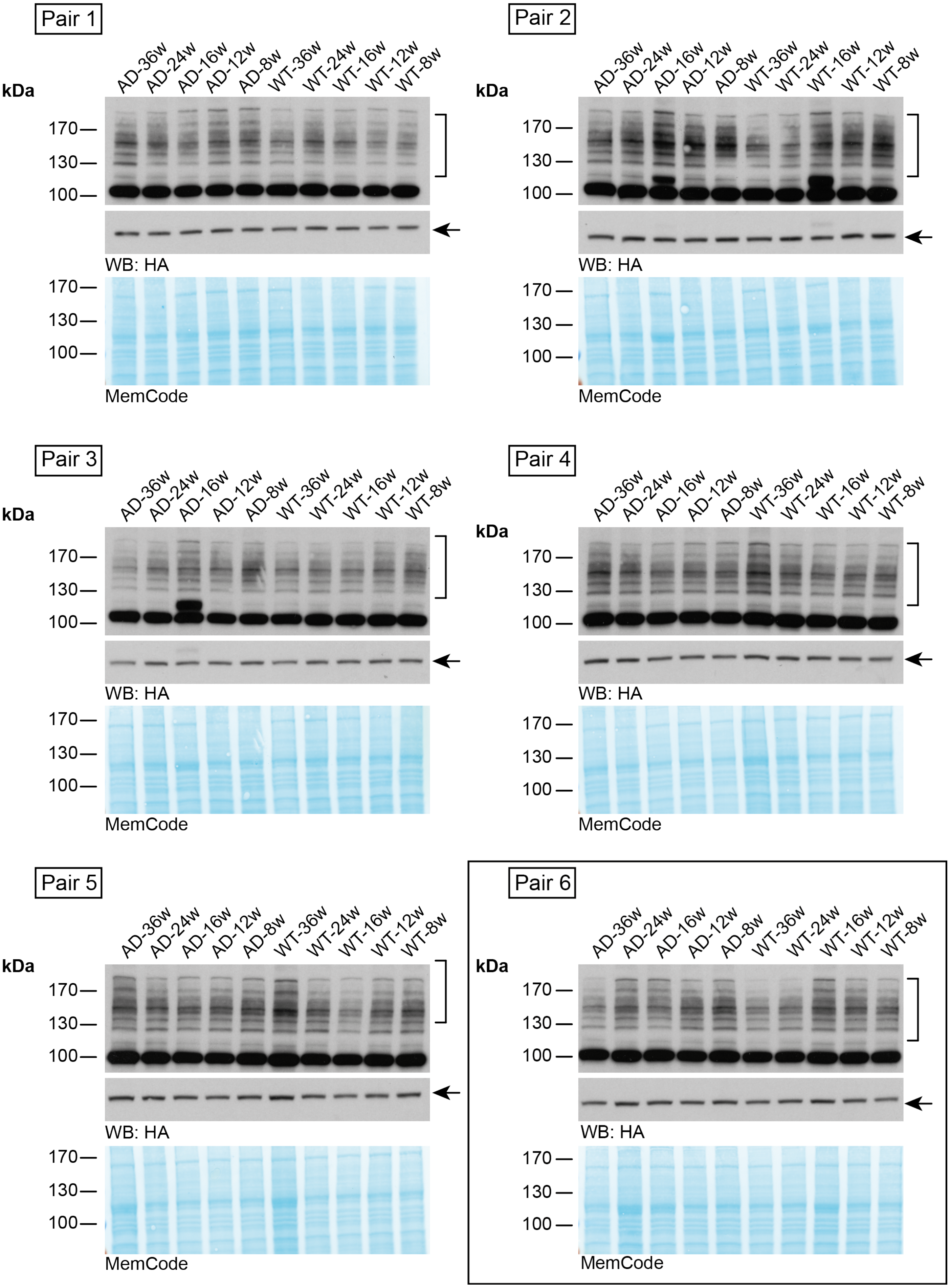

Hippocampal Loading III

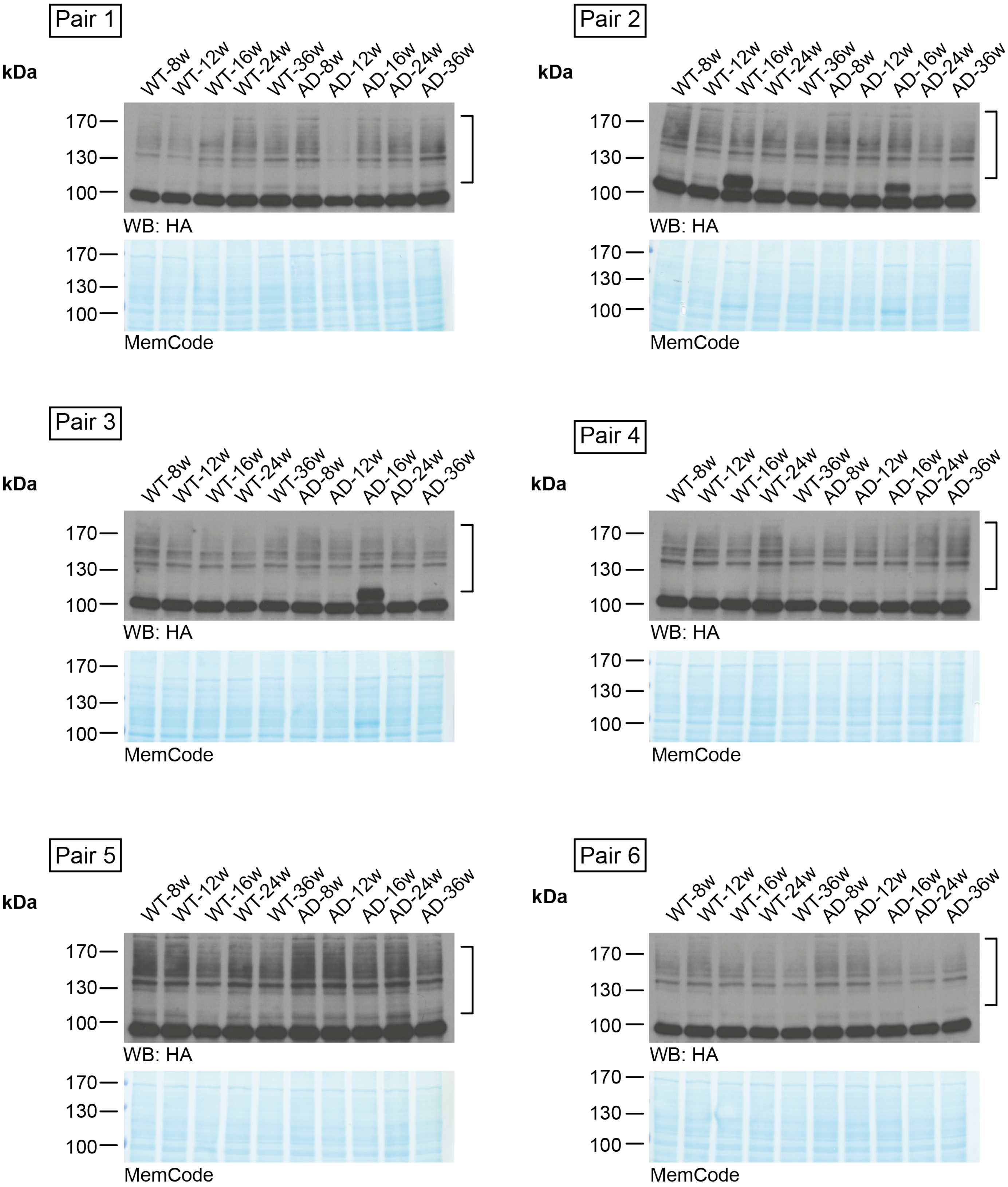

Cortical Loading I

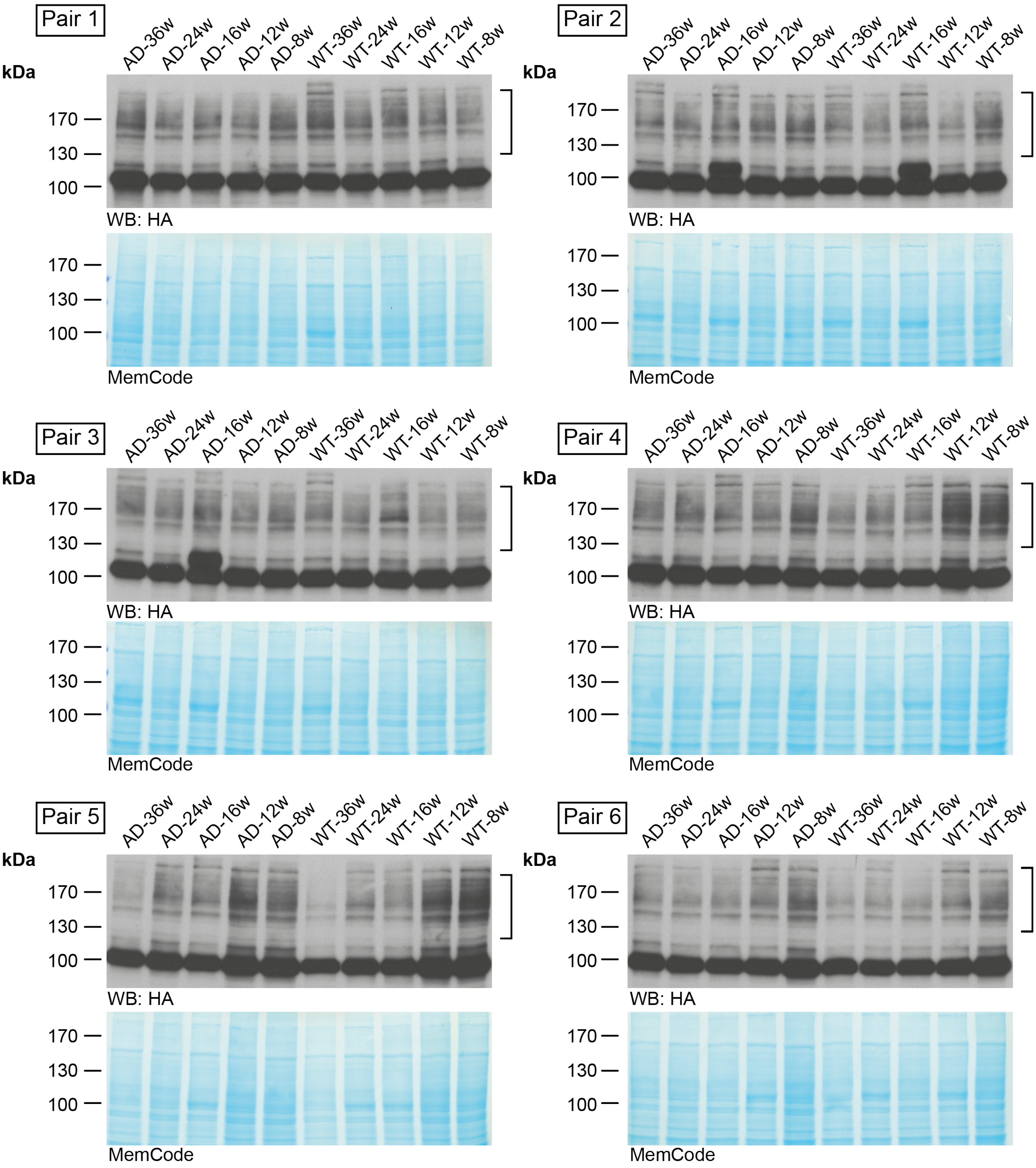

Cortical Loading II

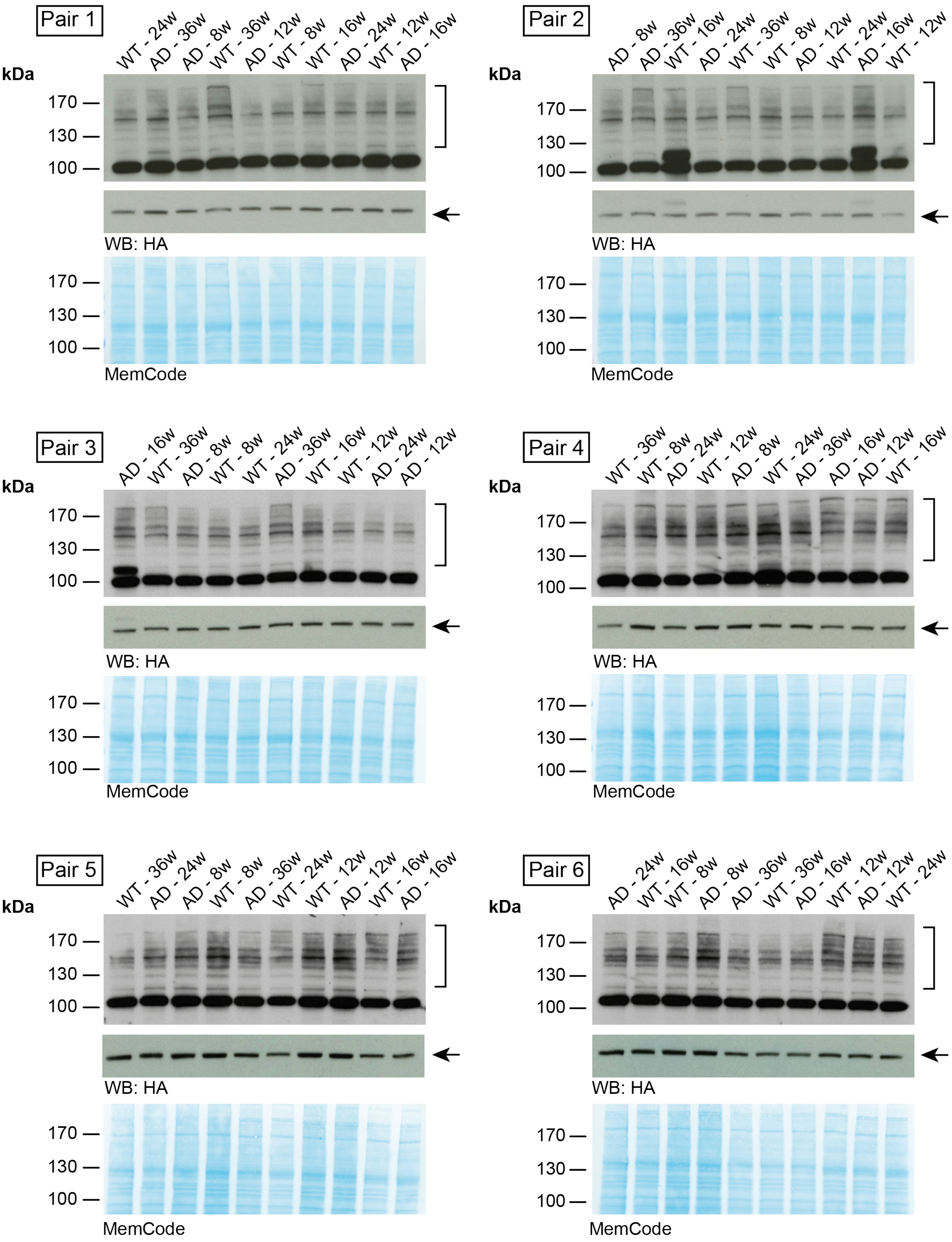

Cortical Loading III

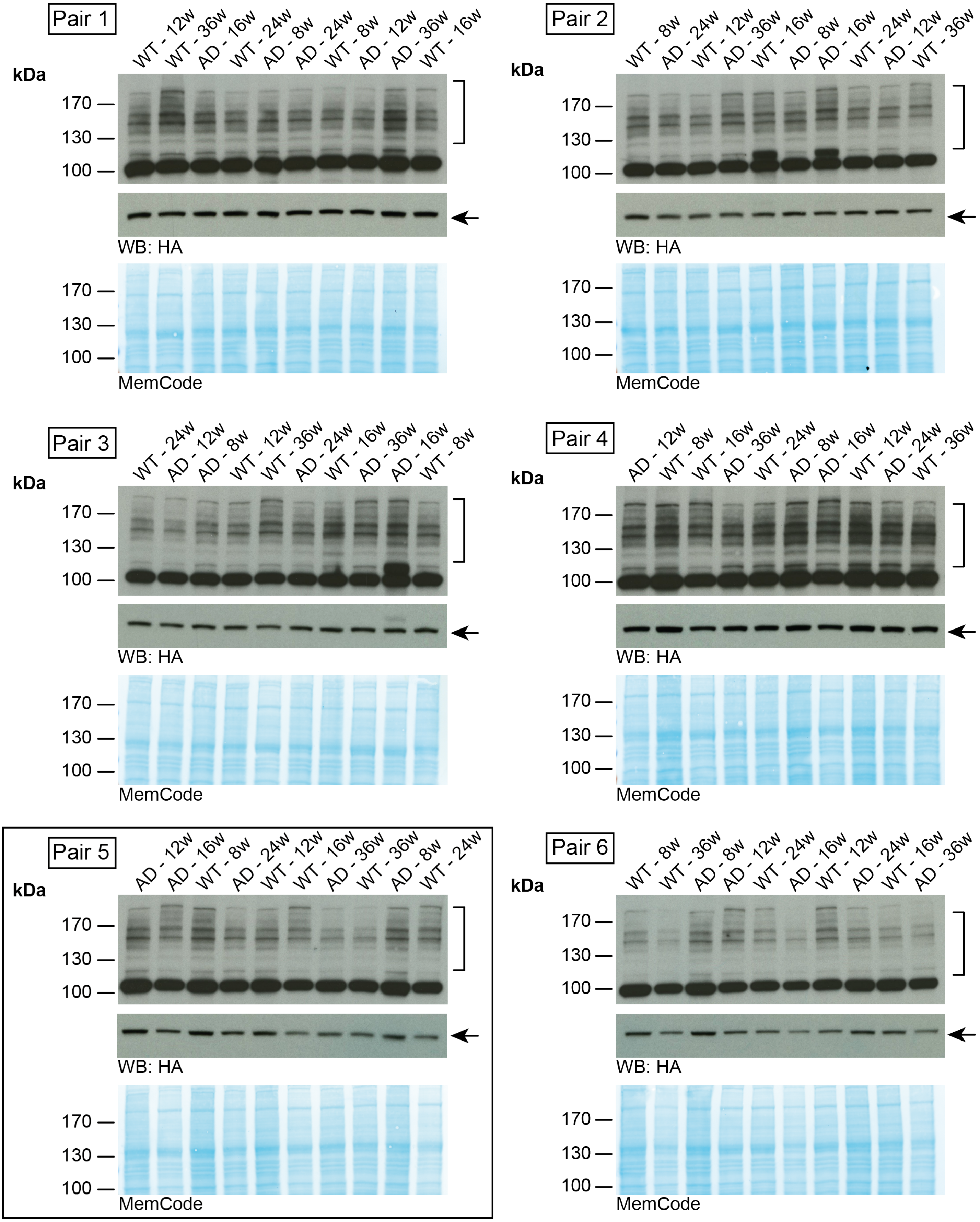

Supplement: Supplementary file 5 [file ACEL-17-na-s005.pdf]
